# Artificial Intelligence Techniques in Grapevine Research: A Comparative Study with an Extensive Review of Datasets, Diseases, and Techniques Evaluation

**DOI:** 10.3390/s24196211

**Published:** 2024-09-25

**Authors:** Paraskevi Gatou, Xanthi Tsiara, Alexandros Spitalas, Spyros Sioutas, Gerasimos Vonitsanos

**Affiliations:** Computer Engineering and Informatics Department, University of Patras, Panepistimioupoli, 26504 Rio, Achaia, Greece; pgatou@ceid.upatras.gr (P.G.); st1067189@ceid.upatras.gr (X.T.);

**Keywords:** artificial intelligence, machine learning, grapevine, diseases, vineyards, smart sensors, smart agriculture

## Abstract

In the last few years, the agricultural field has undergone a digital transformation, incorporating artificial intelligence systems to make good employment of the growing volume of data from various sources and derive value from it. Within artificial intelligence, Machine Learning is a powerful tool for confronting the numerous challenges of developing knowledge-based farming systems. This study aims to comprehensively review the current scientific literature from 2017 to 2023, emphasizing Machine Learning in agriculture, especially viticulture, to detect and predict grape infections. Most of these studies (88%) were conducted within the last five years. A variety of Machine Learning algorithms were used, with those belonging to the Neural Networks (especially Convolutional Neural Networks) standing out as having the best results most of the time. Out of the list of diseases, the ones most researched were Grapevine Yellow, Flavescence Dorée, Esca, Downy mildew, Leafroll, Pierce’s, and Root Rot. Also, some other fields were studied, namely Water Management, plant deficiencies, and classification. Because of the difficulty of the topic, we collected all datasets that were available about grapevines, and we described each dataset with the type of data (e.g., statistical, images, type of images), along with the number of images where they were mentioned. This work provides a unique source of information for a general audience comprising AI researchers, agricultural scientists, wine grape growers, and policymakers. Among others, its outcomes could be effective in curbing diseases in viticulture, which in turn will drive sustainable gains and boost success. Additionally, it could help build resilience in related farming industries such as winemaking.

## 1. Introduction

In the last few years, there has been a major increase in population growth, with more diverse dietary choices and a rapid switch to a more healthy lifestyle. These social transitions, with climatic change, have a direct impact on the agricultural field, which has to overcome these trends by increasing production but also by increasing quality. In this respect, smart farming and precision agriculture try to assist the farmer and modernize agriculture.

Vine production is important for agriculture because of its long history and its contribution to the culinary arts, but it comes with several challenges that may affect farmers and consumers. Agricultural challenges are certainly not new to the world; however, they have now taken on a new scope. This is due to everything changing, changeable and unpredictable weather conditions, pests, diseases, and resource constraints, which make growing grapes challenging. Growing vines can have a mainly positive impact on the economic development of a region but also creates certain problems regarding precise management or reproduction in the event of the dying of the plants. Expert advice is very expensive and is also low in accuracy in helping with these issues. The uncertainty of drought, flooding, and pests has farmers at their wit’s end, trying to adapt their techniques to climate change.

The cultivation of vines faces obstacles that need creative solutions, marrying human ingenuity with the power of technology. Machine Learning is one of the technologies that can optimize vine production. Making very accurate predictions for the guidance of farmers’ decisions is achievable, for instance, by using weather and soil condition data combined with image recognition. Detecting disease and infestation early enough may save the entirety of a crop, and proper irrigation and nutrient management with ML recommendations ensure the resources are used in the best possible fashion. Essentially, Machine Learning is the way forward and is positively impacting the face of agriculture in improving the health, productivity, and economic viability of grape growing while reducing pesticide use.

Grapevine research has changed seriously in the past few years with the increased use of AI tools to analyze data and find trends. This review attempts to provide a comprehensive evaluation of AI techniques in the area of grapevine research by comparing the different datasets, diseases, and techniques. Some of the topics that will be included are grapevine diseases, data format techniques, and Machine Learning algorithms from image format to capturing tools like UAVs. It is designed to deduce the most efficient methods in the evaluation of grapevine data by comparing current methodologies and organizing them for future researchers. Similarly, it promotes the study of new methods by supporting the study of previous best-performing algorithms and less researched algorithms in the field of grapevine research. The above is focused on the areas of prediction for grapevine diseases, Water Management techniques, plant nutrition, and grapevine classification, amongst other studies that have already been conducted in recent years using Machine Learning algorithms. This review supplies a valuable resource for grapevine researchers and practitioners interested in incorporating AI techniques into their work.

### Aim of the Study

We aim to consolidate all research works related to the application of Artificial Intelligence in grapevine studies from 2017 to 2023. This includes summarizing the research works made in that period and comparing the various techniques and datasets utilized in each study. This consolidation can serve multiple purposes, including advancing superior and sustainable grapevine management research, which impacts the development of the wine industry and other sectors dependent on grapevines. Specifically, the objectives of our research are highlighted as follows:

- Knowing that the majority of the research (88%) had been performed in the last 5 years, we needed it to be organized in one place where the studies will be compared together.
- Moreover, we organize all the technologies used and the applications of ML in grapevine research and correlate them together. As a result, it becomes more likely that researchers will look at the general picture and all perspectives.
- Knowing the currently studied ML topics in grapevine research and comparing them with the ML topics studied in general agriculture, it is easier to understand the topics that have not been thoroughly studied yet in grapevine research.
- The datasets are organized in the same place so that future researchers can easily utilize them, while hopefully, future works could arise using the same data for comparison reasons.

## 2. Materials and Methods

We conducted a systematic review by carefully selecting the papers to be reviewed and outlining their techniques and methods in tables and an overview for each one, and we also used VOSviewer (Version: 1.6.19 was released on 23 January 2023) [1] to visualize our queries and ratify them. In particular, VOSviewer can generate keyword maps based on shared networks; as a result, it can create maps with multiple items as well as publication maps, country maps, journal maps based on networks (co-citation), and maps with multiple publications. Users can modify the quantity of keywords utilized and eliminate less relevant terms. In brief, VOSviewer software supports data mining, grouping, and the mapping of articles that are retrieved from scientific databases, as mentioned in research [2].

The Google Scholar query that we executed was ALL = ((“Machine Learning” OR “Artificial Intelligence”) AND (“Grapevine” OR “Vineyard”)) AND (year >= 2017-01-01 AND year <= 2023-01-01), and we carefully chose the results because many of them were essentially citing grapevine research but did not analyze them and were not dedicated to it. We then performed the same query on WebOfScience and passed it to VSOviewer. Finally, each paper has been described with a paragraph outlining the major features, and the results are then compared using papers based on their techniques and applications.

In WebOfScience, we had 158 results, and we also changed the year to “al”, and the results increased to 178 (meaning 88% of the research was performed in the last 5 years). Lastly, we passed it to VOSviewer and chose “Title and abstract fields”, “full counting” and at least 8 occurrences in each paper; the result had 56 terms, and we removed irrrelevant items like “number”.

**Figure 1:**
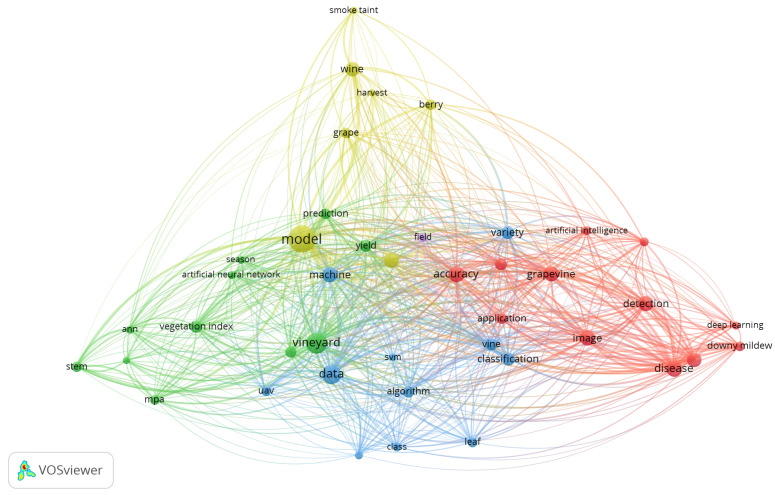
Full network visualization of the scientific literacy topic areas.

**Figure 2:**
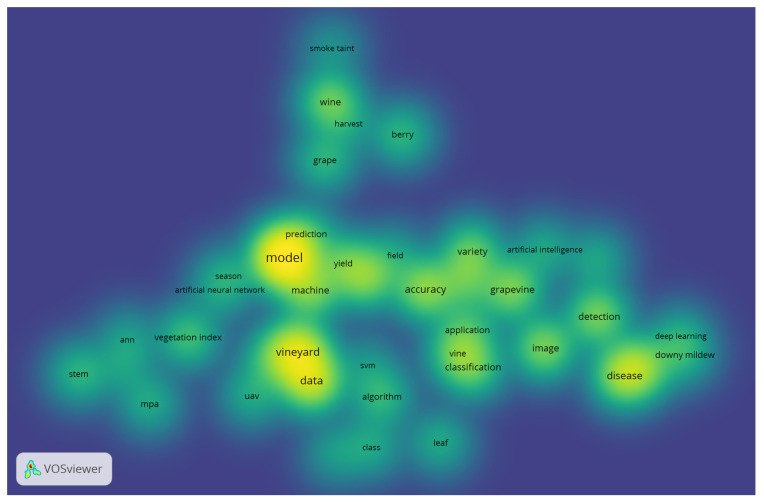
Density visualization of the scientific literacy topic areas.

Topic mapping is vital to bibliometric research [1]. Figure 1 shows all the subject areas about the overall keywords of scientific literacy. VOSviewer can display three different mapping visualizations for the bibliometric analysis, and Figure 2 shows the density visualization.

Figure 2 illustrates the breadth of research on the subject. Essentially, the visualization in Figure 2 serves as a heatmap for unveiling the trends that researchers are working on more frequently. Consequently, based on the findings from the literature, it can be concluded that there is a lot of discussion about the model, vineyard, disease, data, and vine classification. This suggests that between 2017 and 2023, these topics gained the most attention from researchers. Furthermore, nodes or keywords without a network with other keywords could evolve into new study areas.

### Outline of the Paper

The rest of the paper is structured as follows.

In Section 3, the general field of Machine Learning in agriculture is described. Next, in Section 4, an overview of the different diseases that affect grapevines is presented from a biological perspective, while Section 5 includes a variety of grapevine datasets, which are mostly classified depending on diseases. Section 6 gathers a short description of all the techniques utilized in any paper we included in this study. The techniques are divided concerning the capture and modification of the datasets, as well as the ML techniques used to analyze them. In Section 7, all the reviewed papers are briefly summarized and separated by their application in grapevines. Finally, in Section 8, the general deductions of our review are summarized.

## 3. Background

### 3.1. Machine Learning in Agriculture

The need for smart farming is becoming more and more vital as the Earth’s population rises and people try to shift to different and more healthy lifestyle choices. When smart farming reaches each peak, with the use of various machines along with algorithms or systems able to replace or work alongside farmers, the next important movement in agriculture is the use of artificial intelligence to help farmers optimize their outcomes. Many studies have been conducted on the integration of artificial intelligence in agriculture [3,4], as well as several surveys [5,6,7] to compare and describe them compactly as the number of studies in this field is increasing extremely rapidly. In particular, Machine Learning, a subset of artificial intelligence, encompasses various techniques, with Neural Networks being particularly prominent. As has been described in various surveys such as [7], most research is focused on four general fields:

- Crop Management;
- Water Management;
- Soil Management;
- Livestock Management.

In Crop Management, key areas include yield production, disease and weed detection, cultivation recognition, and quality assessment. Comprehensive information on the above can be found in surveys such as [6], which also provide comparative studies in the general problem of different ML applications in agriculture. In this paper, however, we focus on the subcategory of grapevines. In grapevine research, the most studied category is crop management and especially disease detection, as it is the most important field of study, while less studied categories are Soil Management and crop quality.

**Figure 3:**
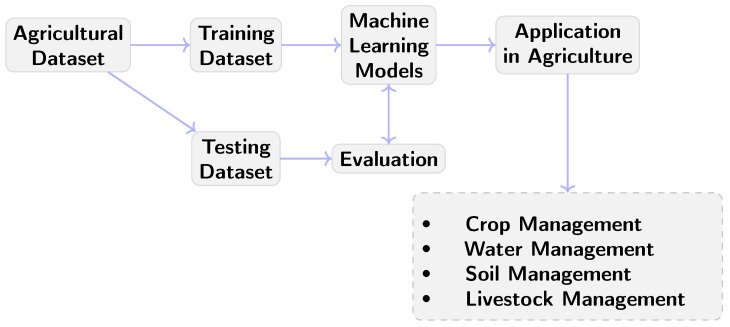
Machine learning process in agriculture (adapted from [8], used under CC BY 4.0, accessed on 11 August 2024).

In general, an ML technique needs four significant components: a good dataset that appropriately describes the general problem, pre-processing of those data for better applicability, a learning phase using the appropriate algorithms that fit the problem, and a testing phase. Figure 3 depicts a common procedure for developing and deploying Machine Learning models in agriculture. The first phase is retrieving agricultural data from a variety of sources, which serve as the foundation for later Machine Learning procedures. The data are then separated into “training” and “testing” datasets. The training dataset is used for training the ML model, whereas the testing dataset acts as an assessment procedure, analyzing the model’s performance and ensuring validity and dependability. These steps result in a strong ML model that can make classifications, predictions, or decisions based on unique agricultural occasions. Afterward, the validated model is ready for use in a variety of agricultural domains, including crop (i.e., optimizing crop yields and health), water (i.e., ensuring efficient irrigation resource utilization), soil (i.e., maintaining soil health and fertility), and Livestock Management [8].

A significant challenge in this field is the scarcity of open datasets, which often leads researchers to create their own, which can massively affect the accuracy of the designed system. Despite these challenges, advances in remote sensing and data acquisition technologies are improving the availability and quality of agricultural data [7].

### 3.2. A Brief Overview of Machine Learning Types

According to the research, ML may be classified based on the following types of learning [9].

**Figure 4:**
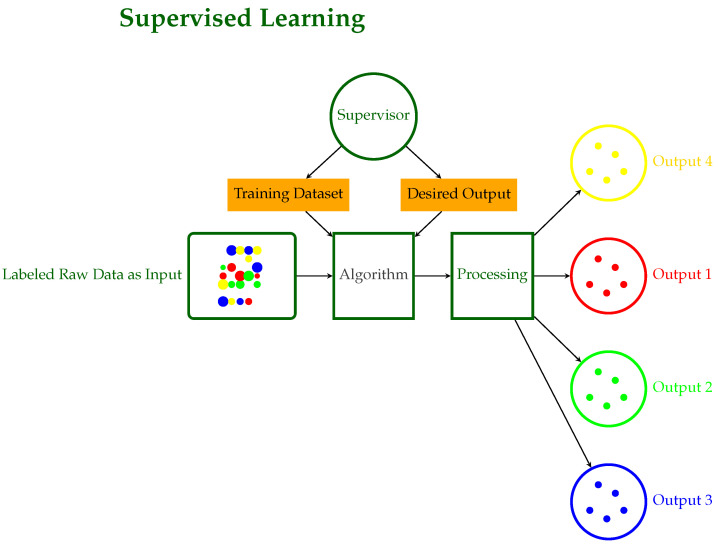
Schematic diagram of supervised learning.

**Figure 5:**
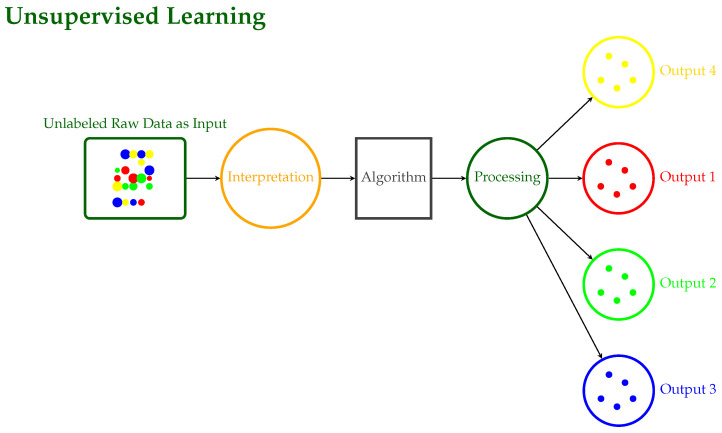
Schematic diagram of Unsupervised Learning.

**Figure 6:**
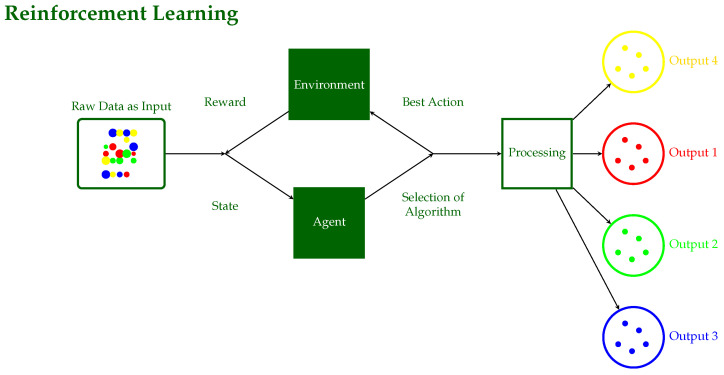
Schematic diagram of reinforcement learning.

1. **Supervised Learning**: The input and output are known, and the machine attempts to find the best path to an output given an input [10]. These models are trained on labeled data and then used to predict future events. The training approach takes as input a known training data set with its associated labels, and the learning procedure develops an inferred function that in turn makes predictions about any new unknown observations that could be added to the model.
  Supervised models are characterized further as regression or classification problems:
  - **Classification**: Classification problems arise when the output variable is categorical, such as “disease” or “no disease”.
  - **Regression**: Regression problems occur when the output variable is a true continuous value such as stock price prediction.
  This family includes models like SVC, LDA, SVR, regression, and Random Forests. Figure 4 illustrates the workflow of a supervised learning system, detailing the key components and their interactions. In this process, an algorithm is trained on labeled data and desired outputs to produce categorized outputs, developing accurate predictions under the guidance of a supervisor.
2. **Unsupervised Learning**: No labels are provided, leaving the learning algorithm to generate structure within its input [6]. It studies the way that computers can infer a function from these data to explain a hidden structure. Rather than anticipating the proper outcome, the system explores the data and may make inferences from them to define hidden patterns in unlabeled data.
  Unsupervised models include clustering and association cases.
  - **Clustering**: A clustering problem is one in which you want to disclose the underlying groupings in the data, such as grouping animals based on particular characteristics/features such as leg count.
  - **Association**: Here, you want to identify association rules, such as “those who buy X also buy Y”.
  Among the models in this family are PCA, K-means, DBSCAN, and mixed models. The corresponding schematic diagram of Unsupervised Learning is illustrated in Figure 5, where raw data are processed through interpretation with successive algorithmic steps to produce multiple outputs without prior training or labeled data.
3. **Semi-Supervised Learning**: A mixture of labeled and unlabeled data constitute the input data [11,12]. This group lies between supervised and unsupervised learning. During model training, a small number of labeled data are combined with a large number of unlabeled data. As in supervised learning, the goal of the system in semi-supervised learning is to train a function such that the output variable can be accurately predicted as a function of the input variables. In contrast to supervised learning, the system is trained on a dataset consisting of both labeled and unlabeled data. Semi-supervised learning is especially applicable when the volume of unlabeled data is huge and cannot be labeled due to either cost issues or difficulty [13].
4. **Reinforcement Learning**: Decisions are made to find out actions that can lead to a more positive outcome, while it is solely determined by the trial and error method and a delayed outcome [7]. Reinforcement learning consists of algorithms that use estimated errors as incentives or penalties. If the mistake is serious, the penalty is harsh and the reward is insignificant. When the fault is little, the penalty is mild and the reward is significant. The two most important characteristics of reinforcement learning are the trial-and-error search and delayed reward. This model family automates the determination of optimal behavior within a given environment to achieve the desired performance. To learn which behavior is ideal, the model requires reward feedback, often known as “the reinforcement signal”.
  This family of models includes the Q-learning, Sarsa, and Markov Decision models. Figure 6 illustrates the reinforcement learning process, where an agent learns to interact with an environment in a way to choose the best actions to obtain desired outputs, take actions, and obtain rewards.

## 4. Diseases in Grapevines

Grape plants are generally affected by infections caused by bacteria, viruses, nematodes, and parasites worldwide [14]. The most common diseases are black rot, mold, yellow spot, and Esca. Early diagnosis and treatment are necessary for such infections. Grapes, leaves, and fruits are generally infected by bacterial, infectious, and viral diseases. Fungal infections are generally judged by appearance, but the most common are bacterial infections. Bacteria generally reproduce by binary fission, whereby one cell divides into two. Black spots, rot, and blight are some of the general diseases found on grape leaves. The researchers approach disease management with generally applicable solutions and specific treatments. Some seek general strategies that can be used across many diseases, while others focus on treating individual diseases. Additional studies were conducted for each disease listed below.

Research shows that the scope of the diseases is quite far and may have an impact on the world economy on a large scale. However, the wide application of pesticides while dealing with crop diseases brings about another issue: the health of those living near the places where these pesticides are applied. The technological advancement of remote sensing data, for example, hyperspectral and drones on multiphotonic sensors, allows catching and monitoring rapid outbreaks of vineyard diseases (e.g., [15]). For instance, an expert lacks the practice precision that could protect or mitigate this transmission of disease. Using scientific research, technological advancement and precise disease control tools are thought of as the driving forces for the provision of the finest vineyard services around the globe.

### 4.1. Grapevine Yellow (GY)

**Figure 7:**
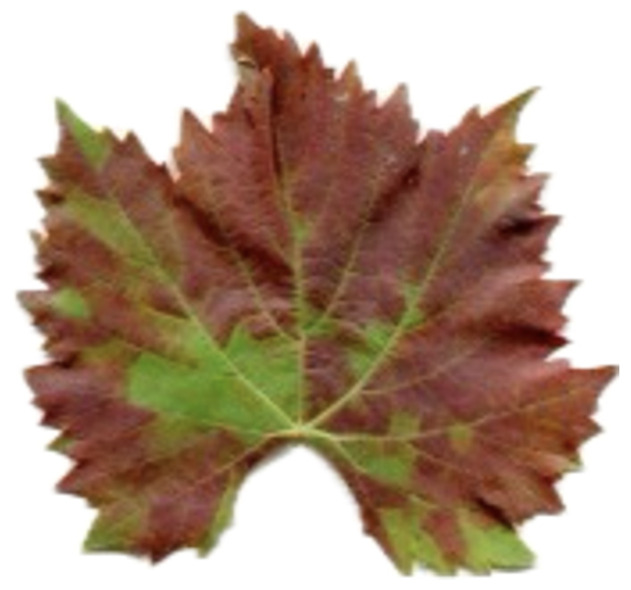
Grapevine Yellow disease (cropped from [18], used under CC BY 4.0).

A variety of Grapevine Yellow [16] diseases are associated with grape growing sites across the world [17], an example is shown in Figure 7. The main varieties include Flavescence dorée (FD), Bois noir (BN), and Palatinate Grapevine Yellows (PGY). These diseases are numerous and caused by a variety of phytoplasma groups. The phytoplasma Candidatus Phytoplasma solani causes BN infection, which is seldom spread by the planthopper Hyalesthes obsoletus. On the other hand, PGY is a health problem related to the Elm yellows group, and the majority of them are transmitted by the leafhopper Oncopsis alni. Candidatus Phytoplasma vitis distinguishes FD, which is spread via Scaphoideus tetanus, a leafhopper. These diseases have similar symptoms, such as the desiccation of flowers, stunted growth, the uneven ripening of wood berries, wrinkled leaves, and discolorations indicative of specific varieties. Leaves can change their colors, from chlorotic yellow to red and purple-reddish, depending on the varietal of the grape. There can also be non- or partial woodiness (at the end of summer) and black pustules on the interveinal portions of the stems. One of the primary reasons we may consider “Grapevine Yellow disease” to be present is because it can cause various manifestations and symptoms that are very similar to those of the other grapevine disease(s), such as leaf roll and/or injury created by leafhopper. Leaf problems can also have colors that are similar to Esca and grapevine leafroll diseases. The phenomenal analysis of Grapevine Yellow disease diagnoses by specialists is the way to obtain the correct results.

#### Flavescence Dorée

**Figure 8:**
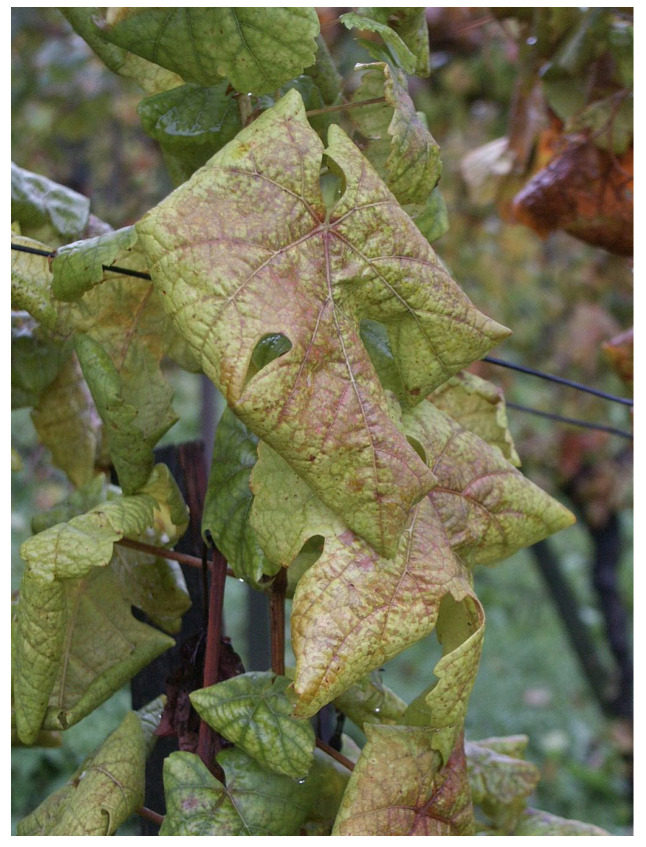
Flavescence Dorée disease (reproduced from [20], used under CC BY 4.0).

Flavescence Dorée (FD) is a specific type of Grapevine Yellow and is usually caused by a bacterial infection that causes it to turn yellow, and the same cause is restricted by the Phytoplasma group of bacteria, an example is shown in Figure 8. This bacteria is being carried by Scaphoideus tetanus, a pest that is transferring bacterium through its bloodstained saliva. FD has an economically important effect in wine-growing countries [19,20], and the emergence of the condition should be expected, caused by the time delay and individuality manifestation. Symptoms of FD comprise dull yellow on white varieties, rubra-red on red varieties, defective lignification on young shoots, flower wilting, fruit drop, and weak shoot development. The problem is that FD symptoms are very similar to those of other grape-yellow viruses such as Bois noirs (BN), so it is easy to mistake them. PCR-based chemical ways of discrimination between typhus and Bacillus Nigra are possible. The present control means the use of measures such as trench digging and the use of pesticides that are harmful to the environment. Earlier detection approaches are mandatory for highly efficient prevention and containment strategies.

### 4.2. Esca

**Figure 9:**
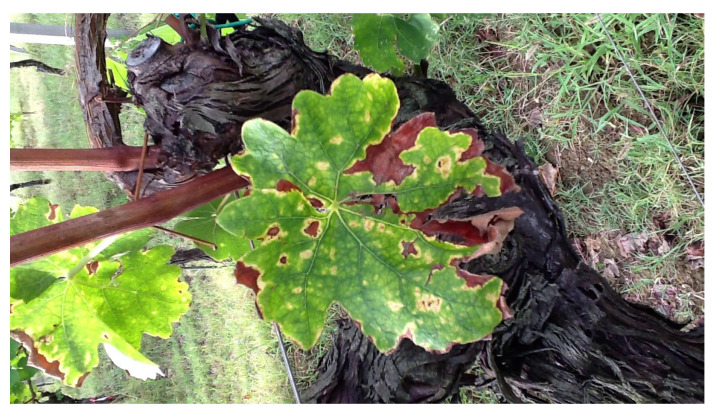
Esca disease (reproduced from [22], used under CC BY 4.0).

Grapevine trunk diseases (GTDs) [21,22] are a highly dangerous threat to viticulture, and Esca is the most harmful disease among them, an example is shown in Figure 9. Esca, as seen normally, results in major economic losses all over the world, and dealing with this problem is further complicated by the insufficient availability of both preventive and curative therapies. GTDs include a complex group of fungi causing mildew and rot such as Eutypa and Botryosphaeria that infect grapes through cuts in the field and from nurseries. The transmission of the GTDs by offshoots from young grafted vines is also possible. There is no type of vine of either the cultivated or wild type that cannot be affected by this disease. The usual Esca wood that affects the old vines shows itself in various discolorations, necrosis processes, vascular infections, and white rot. Conversely, it is the outward symptoms that later appear, which may take months and may appear years after infection due to the slowness and slow progression of the illness. Often, the plants look sick or have chronic intervascular necrosis, yellow or red chlorosis, and tiger stripes on leaves or shoots. The degree of tree symptoms comes short of the sign of leaf symptoms in certain cases. Since chronic symptoms do not develop consistently, annual monitoring is crucial for accurately assessing the disease’s incidence in a vineyard.

### 4.3. Downy Mildew

**Figure 10:**
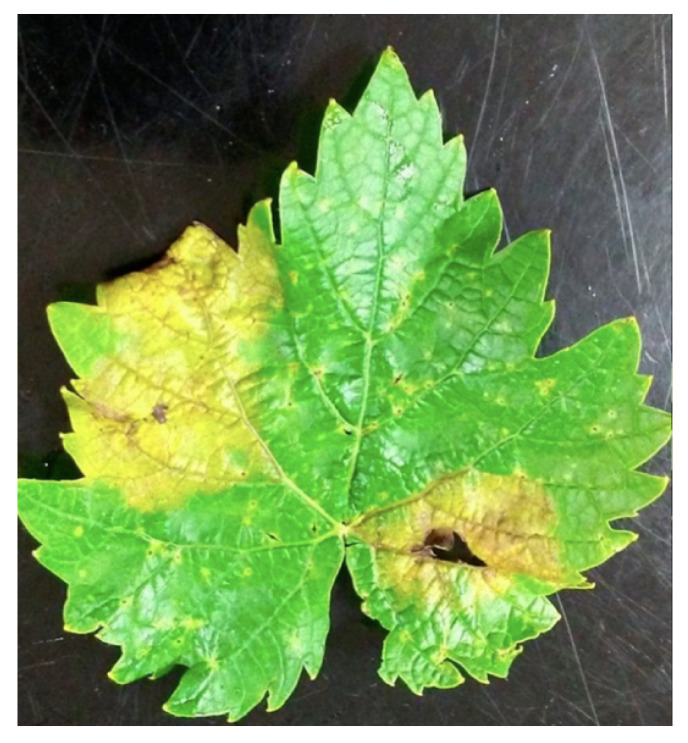
Downy mildew disease (cropped from [24], used under CC BY 4.0).

Downy mildew [23,24], known as a disease of grapevines caused by the oomycete pathogen Plasmopara viticola, is one of the major diseases, an example is shown in Figure 10. It is well known to result in a heavy harvest reduction, in addition to affecting the quality of fruit during several growing seasons. The disease was coincidentally transmitted to Europe towards the end of the 19th century and has since resulted in a destructive effect on grape yields. In the primary phase, the germinated oospores develop into macrosporangia in the spring, and then they begin to release zoospores under moist conditions. The early stages of downy mildew infection are followed by boosting the transpiration coefficient and a decrease in leaf temperature. During the later stages of the disease, the bark turns chlorotic and necrotic, causing moisture loss and reduced stomata control. Treatments with fungicides are employed to stop downy mildew of the grapevines, but excess fungicide application is not economical, environmentally friendly, or safe for human health. In the case of downy mildew identification, it is necessary to distinguish its symptoms from the other pests or missing symptoms. In the early stage of the infection, small yellowish-brownish oil patches may occur on the leaves of grapevines, which can resemble symptoms caused by pests, such as spider mites. The increasing growth of spider mites usually occurs in the form of yellow or red strokes along the veins of the leaves. Distinguishing between the types of yellowing is a key feature for a diagnostician who is trying to diagnose the cause of aging correctly and provide proper control measures. An adequate diagnosis gives commercial vineyards a chance to tailor specific pest (spider mite) or disease (downy mildew) control interventions or explore other options.

### 4.4. Leafroll

**Figure 11:**
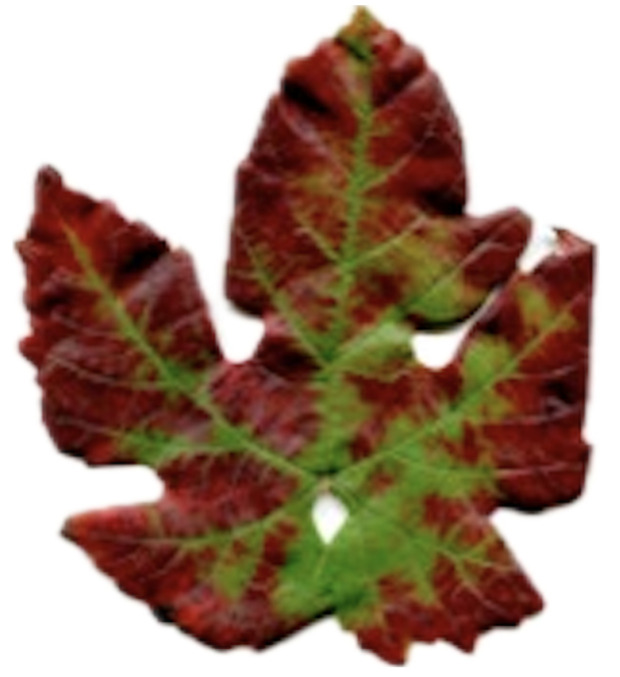
Leafroll disease (cropped from [18], used under CC BY 4.0).

Grapevine leafroll disease (GLD) [18,25] is a viral disease that negatively impacts wine grape production worldwide, an example is shown in Figure 11. It impacts grapevine vigor, physiology, and the final grape quality. GDL is subject to the following problems: ripening is uneven, and bunches bear less fruit with lower sugar content. Several viral species of grapevine leafroll-associated virus (GLRaV) are being identified in grapevines, such as GLRaV-3, the most prominent and the main pathogen of grapevine leafroll disease. When herbicides penetrate the leaves, photosynthesis is reduced, and that may impair the level of chlorophyll and carotenoids [26]. This slowdown in photosynthetic processes can be a critical step in the weakening of the overall health and productivity of the vine. The economic consequence concerning GLD, including other well-known virus diseases such as the Grapevine Leafroll-associated Virus Complex 3 (GLRaV-3), is huge, which leads to a great number of revenue losses of billions of USD annually within the US wine and grape industry. Amongst the GLD management and control measures are items like using virus-free planting material, exercising correct sanitation measures, and improving the cultural practices that minimize the spread of the disease. An early diagnosis of GLD and the identification of a spectrum of infectious viral species is paramount for the implementation of functioning management in a vineyard.

### 4.5. Pierce’s Disease

**Figure 12:**
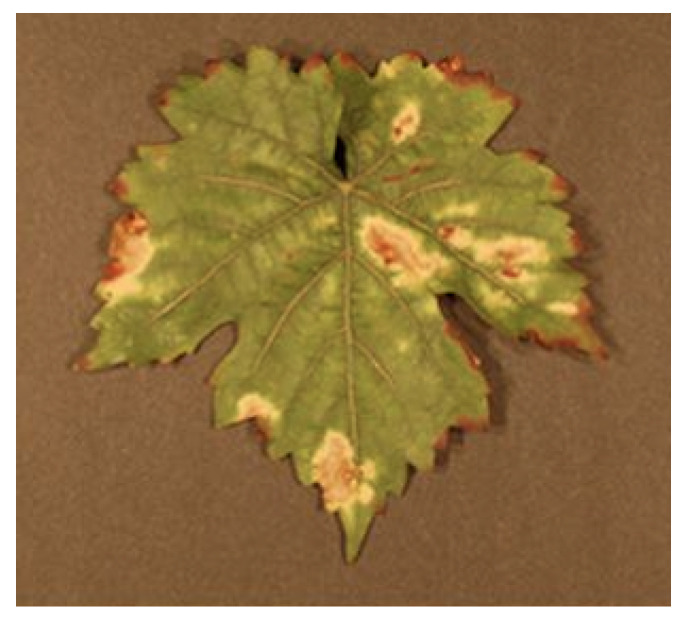
Pierce’s disease (cropped from [29], used under CC BY 4.0).

Pierce’s disease (PD) [27] has become a major threat to wine grapes, especially in the US and specifically California, an example is shown in Figure 12. Downy mildew is very deadly, as there is no cure for it and diseased grapevines can die from it in five years. PD is found to have a significant economic impact on the same measures, yielding huge losses that could overpass the 100 million dollars for the state of California annually. PD is a disease that, if not found and discovered on time, can affect commodity grape productions hence the need to continuously find a PD throughout. The PD manifestations may be observed 3–6 months and up to 18 months after the infection has started. They give signals that tell whether there is any danger or not. In contrast, one can confuse the powdery mildew’s symptoms with those of some other vine diseases and disorders, so exact diagnosis is vitally required. The earliest detection of PD is advisable for the application of necessary management strategies and thus, prevents the disease from becoming more severe. Among other economic consequences of the loss of grape and wine PD by California State grape and table grape growers, this total has dropped dramatically by about 104%. The number was 4 billion in 2014, and in 2018, it increased dramatically to 5.6 billion. Many preventive activities are being carried out to mitigate this problem and find appropriate solutions to maintain good health, which in turn ensures the sustainability of viticultural activities [28,29].

### 4.6. Root Rot

**Figure 13:**
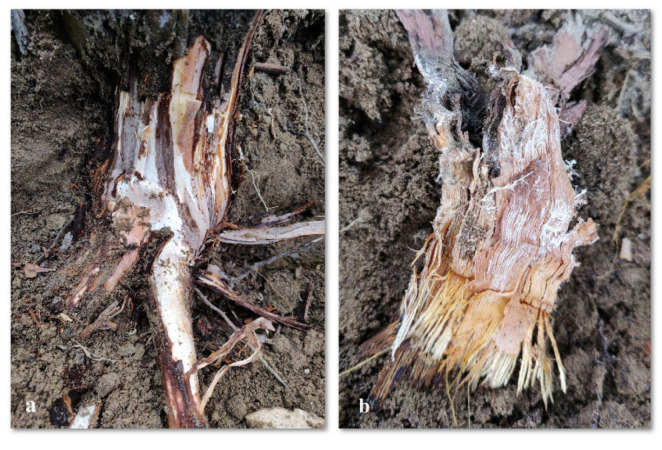
Armillaria Root Rot disease (reproduced from [31], used under CC BY 4.0).

Another elm pathogen is Armillaria Root Rot [30,31] which is an ubiquitous, chronic problem for ornamental and other woody plants, including grapevines, an example is shown in Figure 13. Recognizing plants infected in the earliest stages is a principal step in the right handling of the pathogen. Across the globe, among several pathogenic fungi, the Armillaria is the most studied and analyzed genus. The contamination is transported via the soil particles, mainly by way of the rhizomes or specialized hypha commonly called rhizomorphs. They are the fastest-growing living bridges among existing fungi in the world, up to 100 m long or more and can penetrate the phloem of host plants. Armillaria molds are a group of opportunistic parasites, some of which are regarded by many primary pathogens of stressed trees, like in the case of changing climate conditions. The pervasiveness of monocultures and the intensification of crop cultivation provide a suitable occupational niche for Armillaria, the fungus known to cause considerable yield losses. Many plants undergo contamination like larch, spruce, pine, fir, or other conifers as well as broadleaf trees, garden trees, shrubs, and fruit bushes including apples, blueberries, pears, peaches, and kiwifruit, which may be infected by Armillaria. It is very difficult to diagnose Honey mouth stem rot just based on foliar population, as they give a general kind of symptoms. Visual studies of stem bases will help doctors to improve their diagnostic imaging. Nevertheless, the above-ground symptomatology of the disorder commonly becomes noticeable at the later or terminal stages when the host plant has lost a significant proportion of its health. A sensitive and speedy early identification is a must to devise the right control measures for the abatement of Armillaria Root Rot in grapevines and other plants.

## 5. Datasets

From our study, we acknowledge the difficulty of finding datasets about vineyards, which led many researchers to create their own datasets. To address this issue, we collected all datasets we could find, and we describe them in the next paragraphs. They consisted mostly of datasets mentioned in the papers we reviewed, but we also researched on our own to have an overview of the field. Next, we describe each dataset by its data type (e.g., statistical, images, and type of images) and the number of images existing in each category (if it is mentioned).

1. GrapeCS-ML database: [32], https://researchoutput.csu.edu.au/en/datasets/grape-image-database (accessed on 10 May 2023). The GrapeCS-ML database consists of images of grape varieties at different stages of development, together with the corresponding ground truth data (e.g., pH and Brix) obtained from chemical analysis. The database consists of five datasets for 15 grape varieties taken at several stages of development and includes size and/or Macbeth color references. Altogether, the database contains a total of 2078 images.
2. In the paper “LDD: A Dataset for Grape Diseases Object Detection and Instance Segmentation” [33], https://www.kaggle.com/datasets/piyushmishra1999/plantvillage-grape (accessed on 10 May 2023), the authors created a grapevine disease database with images from Horta’s internal databases, the competition Grapevine Disease Images, and the web, as well as manual segmentations. It contains 1092 RGB images of grapes and 17,706 annotations (instances) for the tasks of Object Detection and Instance Segmentation. More specifically, it contains the categories black rot with 1180 instances, Esca (black measles) with 1383 instances, leaf blight (Isariopsis leaf spot) with 1076 instances, and healthy with 423 instances.
3. Grapes-Leaf-Disease-detection repository, https://github.com/shreyansh-kothari/Grapes-Leaf-Disease-detection (accessed on 16 May 2023), consists of images about grapevine diseases. To be exact, it includes Esca (240 images), black rot (210 images), Healthy (220 images), and leaf blight (210 images).
4. In [34], https://github.com/cu-cairlab/iros2022-OnlineDMSeg.git (accessed on 18 May 2023), there is a dataset with 282 raw images, classifying them as healthy or with downy mildew disease.
5. The paper [18] includes a collection of datasets augmented with the plantVillage dataset for grapevine diseases. It consists of the categories black rot (1180 images), Esca (1383 images), Grapevine Yellow (134 images), healthy (84 images), and leaf blight (1076 images). They also have edited versions of the images with various operations, increasing the total number of them. These datasets can be found on the links below (accessed on 25 May 2023):
  - https://plantvillage.psu.edu/
  - https://www.kaggle.com/datasets/mohitsingh1804/plantvillage
  - https://github.com/DrAlbertCruz/Salento-Grapevine-Yellows-Dataset
6. This dataset [22], http://dx.doi.org/10.17632/89cnxc58kj.1 (accessed on 2 June 2023), is specific to Esca disease in grapes and consists of 881 healthy RGB images and 887 Esca images.
7. For the paper [35], https://www.kaggle.com/datasets/muratkokludataset/grapevine-leaves-image-dataset (accessed on 8 June 2023), they also created a dataset using hyperspectral imaging under laboratory lighting. their dataset consisted of 496 images of 248 plants. Also, this dataset distinguishes between symptomatic and asymptomatic leaves.
  The images in the collection were captured on grapevine leaves from five different varieties: Ak, Ala Idris, Büzgülü, Dimnit, and Nazli. Each kind of grapevine species has 100 images. The same dataset was used in [36].
8. Some research used also statistical data for grapevine disease (e.g., [37]); some statistical data for diseases can be found on the following page based on grapevines located in Italy: https://agroambiente.info.regione.toscana.it/ (accessed on 13 June 2023).
9. The paper “An expertized grapevine disease image database including five grape varieties focused on Flavescence dorée and its confounding diseases, biotic and abiotic stresses” [38], https://data.mendeley.com/datasets/3dr9r3w3jn/2 (accessed on 7 August 2024), contains a dataset that has 1483 RGB images of five different grape varieties, and two different diseases (Doree and Esca). This dataset can be used both for classification purposes of the grape varieties, as well as for disease detection. The images were acquired during 2 years from 14 vineyard blocks located in France.

The previously described datasets are compared in Table 1. This table provides an overview of different datasets used in reviewed grapevine disease detection and classification studies. Nine different datasets are listed, each designated with a number from 1 to 9, referencing the datasets mentioned above.

The table lists specific diseases affecting grapevines in “DISEASES” columns, including “GY” (Grapevine Yellow), “Esca”, “Mildew” and “Leafroll”. The “Others” column includes other, less common diseases. The numbers in each cell indicate the number of raw images used for each disease within the corresponding dataset. Some datasets include multiple diseases; for example, Dataset 5, which is an expansion of Dataset 2, has 134 images for Grapevine Yellow (GY), 1383 images for Esca, 1180 for black rot, and 1076 for leaf blight, summing to 3773 total images related to these diseases.

The “CLASS” column indicates the total number of image data points used for classification purposes in each dataset. For example, Dataset 1 contains 2078 images used to classify grape varieties and chemical data.

The “INFO” column provides additional context or specific notes about each dataset, highlighting the inclusion of diverse data types, such as chemical and statistical data, alongside images, suggesting a multifaceted approach to grapevine health analysis.

Overall, the table offers a detailed breakdown of the datasets used in the study, emphasizing the variety and specificity of the data collected to support Machine Learning applications in grapevine disease management.

## 6. Techniques in Grapevines

### 6.1. Data Format Techniques

In this subsection, we briefly introduce the techniques used in the data aspect, so we can concentrate on how the data can be captured and in what form.

Also, in Table 2, we summarize the data format techniques used in various studies related to grapevine applications, categorized by different tasks such as disease detection, water status monitoring, and plant deficiencies, along with the relevant notes. This table highlights the diversity of data acquisition techniques employed in grapevine research. As demonstrated, spectral and thermal imaging, along with UAVs, are prominently used for disease detection and Water Management. RGB imaging remains a foundational technique across all the aforementioned applications.

#### 6.1.1. RGB Images

One of the most common options for Machine Learning applications for capturing detailed color information and providing rich visual data is RGB images [68]. The RGB color model is a way of displaying colors in images using red, green, and blue as the three primary colors in the additive color system and represents a wide range of colors using different intensities. Therefore, RGB images are well established as a way to capture image data for disease detection and plant species distinction. This digital image analysis is a useful and non-destructive method. The RGB system is representative of high spatial resolution with limited spectral analysis. In spatial analysis, we can have pixel resolutions where the order is a few millimeters or smaller and it can be performed in separate measurements. Afterward, features like morphology or texture can be extracted from the digital image analysis. Texture refers to visual patterns and the spatial arrangement of the pixels that constitute a photo. Using texture, we gain more information than can be obtained from color or intensity analysis separately. The variation and the relationship between the pixels that form the surface and the structure of the objects in the image can be captured from the texture. So, the objects can easily be separated and identified. For instance, in grapevine disease detection, texture can detect different plant differentiations that are related to the disease symptoms like spots and discoloration. Thus, we will have a more accurate prediction [69].

#### 6.1.2. Spectroscopy

Spectroscopy is a technique used to analyze the interaction between matter and electromagnetic radiation. It acquires information over a broad spectrum range, detecting vibrations at certain frequencies corresponding to bonds or a group’s transition energy. Spectral resolution refers to the spectral measurement bandwidth. The spectral analysis systems have narrow measurement bandwidth; hence, they resolve spectral features to very high quality. One of the noninvasive sensing technologies is visible/infrared spectroscopy, which, by spectral information in the VIS or IR spectral range, opens up the rapid determination of the objects without sample preparation. The VIS region is a portion of the electromagnetic spectrum visibly perceived by the human eye and contains information about color features we can perceive. IR falls between visible and microwave regions of the electromagnetic spectrum and hence is not visible to the human eye. The IR region contains information about chemical compounds and their structures. This spectrum is divided into three main regions: Near Infrared (NIR), Mid-Infrared (MIR), and Far-Infrared (FIR). The NIR is beyond the visible spectrum, can activate overtones or harmonic vibrations, and offers information undetectable to the human eye. NIR spectroscopy finds its uses in agricultural monitoring, like evaluating plant health, soil properties, etc. Next, MIR is beyond the NIR and is associated with basic and rotational vibration structures. It comprises information related to chemical functional groups, such as the detection of chemical changes associated with diseases. It is utilized in, for example, natural and biomedical applications. FIR is just before the microwave region, is related to warmth radiation, and is generally utilized for thermal imaging and temperature measurement applications [69]. Furthermore, the Short-Wave Infrared (SWIR) is a portion of the infrared spectrum that is among the regions of NIR and MIR. Its uses include the monitoring of vegetation health, soil moisture, and chemical analysis. Moreover, the Visible and Near-Infrared (VNIR) portion is a combined spectral range that shares the spectrum of VIS and NIR. This spectral range can be used in both hyperspectral imaging and environmental monitoring. VNIR sensors have valuable use in agricultural management and environmental science [70].

One of the major spectrometers is the Airborne Visible/Infrared Imaging Spectrometer, AVIRIS, designed to extend spectroscopy to Earth remote sensing. It was designed and developed for NASA’s research and its applications. This spectrometer aims to provide access to high-quality spectral images acquired in multitemporal conditions over all regions of the Earth. NASA’s Airborne Visible/Infrared Imaging Spectrometer Next Generation (AVIRIS-NG) is one of AVIRIS’s family, which collects hyperspectral data across both visible and infrared portions of the spectrum. Examples where we can use this spectrometer are the following [71,72].

1. Classification and Mapping: The valuable spectral information gained from the AVIRIS-NG can be used with Machine Learning algorithms for classification and mapping applications. This may include the mapping of vegetation, surface and open water, and crop types. They can also use the labeled AVIRIS-NG data in other images to automatically classify objects or features.
2. Environmental Monitoring: We can use AVIRIS-NG images to estimate environmental parameters such as vegetation health, water quality, pollution, etc. Machine Learning algorithms permit the examination of the spectral patterns and correlations in AVIRIS-NG data for the detection and observation of anomalies or changes over time, which is greatly helpful in identifying environmental hazards or changes in ecosystems.
3. Disease Detection: The early detection of plant diseases is feasible by training Machine Learning models on AVIRIS-NG images. The algorithms detect even minor changes in vegetation health since AVIRIS-NG captures the spectral signatures of vegetation. Thus, farmers and researchers make informed decisions to control the spread of diseases and protect crops.
4. Mineral Exploration: AVIRIS-NG data can be used in Machine Learning applications for mineral exploration and mapping. In the reflectance spectra, there are unique absorption features for rocks and minerals that can be observed from the spectral signatures obtained from the AVIRIS-NG images. Training Machine Learning algorithms may allow one to identify and detect minerals and hence locate and map deposits of various minerals.

#### 6.1.3. Hyperspectral Images

Spectral techniques have been proven to enhance accuracy in many instances and are also quick, nondestructive, repeatable, and economical [73].

Hyperspectral images (HSIs) are useful in Machine Learning and precision agriculture for detecting and monitoring plant diseases. Several congruent images constitute the hyperspectral image data. These images capture a large amount of spectral and spatial information and provide a wide range of wavelengths that the human eye cannot see. Also, they can identify and distinguish spectrally similar materials and compare them with other types of remotely sensed data so that we can extract more accurate and detailed information. These data represent different wavelength bands of vector pixels (voxels) containing two-dimensional spatial and spectral information. Continually, from HSI, we can obtain reflectance values allowing, us to identify anomalies and potential risks to plant development, facilitating the early detection of and reduction in economic impacts. Spectral measurements, including spectral reflectance and vegetation indices, are important in attendance to plant health. They assess biophysical, biochemical, and geometric observations of size orientation, shape color, texture, pigment change, water content, and tissue functionality. They have been evaluated for their potential in detecting and differentiating crop diseases. In conclusion, hyperspectral imaging, spectral measurements, and Machine Learning algorithms provide relevant information for disease detection, monitoring plant health, and implementing variable rate technology in precision agriculture [69].

#### 6.1.4. Multispectral Images

Multispectral imaging captures image data within specific wavelength ranges across the electromagnetic spectrum, such as visible or near-infrared regions. In addition, multispectral imaging measures light in a small number of spectral bands. Each region captures the intensity of radiation within that specific range of wavelengths. We can thereby extract information that the human eye fails to capture. These images can be used in various remote sensing applications like satellite imagery and aerial photography for mapping and analyzing details of the Earth related to landforms, coastal boundaries, vegetation health, and identification of certain features [74].

The main difference between multispectral and hyperspectral images is that multispectral images capture a limited number of spectral bands, while in hyperspectral images, hundreds of continuous spectral bands are captured. Also, hyperspectral images provide more details and exact spectral information, and as a result, the analysis is more accurate. On the other hand, hyperspectral data are computationally demanding and require different, specific algorithms for processing and analysis.

#### 6.1.5. Thermal Images

Infrared thermography (IRT), often known as thermal imaging, is a process where a thermal camera records and produces a picture of an item by utilizing infrared radiation emitted from the object, which is an example of infrared imaging science. It is a useful technique since we can analyze and detect the health of plants. Leaf stomatal closure is related to surface temperature and transpiration. The canopy or leaf temperature can be an indicator of several physiological parameters such as stomatal conductance. Advances in thermal imaging technology offer new ways to explore the plant’s thermal response to water status.

Leaf temperature measured by infrared thermography [75] may be an early measure of crop disease and stress before the appearance of visual symptoms. Among all the electronic imaging sensors, thermal sensors appear to be more efficient than others for detecting disease-related changes. For instance, we can tell that infrared cameras have been used to differentiate between biotic and abiotic stresses in cotton [76]. In leaves, the maximum temperature difference can be increased during pathogenesis. Thermal imaging has also been employed to monitor the horizontal spread of infection in rose and cucumber before any visual symptom development. However, ambient temperature and other environmental variables such as sunlight radiance cause fluctuations in leaf temperature and may obscure the infection-caused changes. Therefore, it is difficult to interpret thermal images and discriminate variations due to infection from those due to environmental variables. Thermal imaging for assessing cotton water status variability was determined midday [77,78].

Overall, infrared thermography offers a fast and non-invasive method for plant health assessment and for detecting stress or infection by measuring leaf surface temperature changes.

#### 6.1.6. Unmanned Aerial Vehicle (UAV) Images

Unmanned Aerial Vehicles (UAVs), commonly known as drones, were originally developed for military missions. Over the years, as technologies improved, their use expanded to many applications such as aerial photography, site surveying, agricultural work, and more. The visible and infrared spectra contribute greatly to disease detection in agriculture, and their combination improves this detection capability according to research that has been carried out. However, the use of two separate sensors for this visible and infrared imaging in UAVs creates a problem, and there is a spatial mismatch therefore the simultaneous processing of data, from both sensors, is difficult. To solve this problem, multimodal alignment or logging techniques can be used to merge the data. These techniques often use Deep Learning methods to achieve this.

In agriculture, UAVs have proven to be valuable tools for many applications. Some of these applications include calculating fertilization rates, monitoring biomass production, and detecting weeds and plant diseases. However, automatic symptom detection remains difficult even though technological advances have been made.

Machine Learning techniques can significantly improve the usefulness of agricultural data obtained from UAVs. For example, to be able to identify specific diseases in crops or detect anomalies in plant health, we can train Deep Learning algorithms based on aerial images captured by UAVs. The algorithms can learn from large data sets, resulting in accurate and efficient disease detection and monitoring. Also, through Machine Learning models, the huge amount of data obtained from UAVs can be analyzed to extract valuable information and patterns to improve decision-making in agriculture [50].

Therefore, this combination of UAVs and Machine Learning has several advantages. First, it offers great potential for increasing productivity, optimizing agricultural practices, and making precise and targeted interventions. Still, farmers and researchers can have timely and high-resolution data at their disposal. This enables them to detect diseases early, monitor crops better, and manage resources more efficiently.

#### 6.1.7. Advantages and Limitations of Data Formatting Techniques

In Table 3, we provide a general overview of the advantages and disadvantages of all data formatting techniques utilized in the reviewed papers. These techniques are arranged in ascending order according to their equipment cost.

RGB imaging [48,69] is a popular choice for visual inspection and basic image analysis due to its accessibility, low cost, and ease of use. It provides high spatial resolution and can be used without specialized equipment, even with smartphones, making it a simple and convenient option. However, RGB imaging is limited to the visible spectrum, meaning it may miss early-stage diseases or subtle chemical changes in plants. It also struggles with asymptomatic or early detection applications due to its limited spectral information.

Thermal imaging [61,78] is an effective method for detecting plant stress and monitoring water status due to its ability to identify temperature anomalies in plants. Its low cost and compatibility with other imaging techniques make it a versatile option for agricultural monitoring. However, thermal imaging is limited to surface temperature data and is highly sensitive to environmental factors such as sunlight and wind, which can distort the accuracy of its readings. As a result, while it adds one more valuable parameter for assessing plant health, it is challenging to use in isolation for applications like disease detection.

UAV imaging [50] enables the efficient coverage of large areas and has the capability to integrate multiple sensor types, such as RGB, thermal, and multispectral, providing comprehensive data for agricultural monitoring, without the need to deploy sensors in every plant. UAVs can be automated, allowing for the more frequent scanning of entire fields, which facilitates the faster detection of problems. Despite these advantages, UAV imaging requires a significant initial investment in equipment and trained operators. Additionally, regulations regarding flight permits vary by country, further complicating the use of UAVs. Moreover, UAVs are limited by their battery life, resulting in restricted flight time, and their performance is highly dependent on weather conditions, which can affect both safety and data quality. It has a high initial cost of investment; however, over time, the cost may decrease as UAV use becomes more frequent and efficient, while it also reduces the need for independent sensors in every plant and automation reduces the cost of workers.

Multispectral imaging [43] achieves a balance between spatial and spectral data, making it a useful tool in various applications. Unlike hyperspectral imaging, it captures fewer spectral bands, which reduces data complexity and makes it more affordable but may miss finer details that could be critical for early disease detection. While multispectral imaging offers comparable results to hyperspectral in many cases, it still involves moderate computational demand and faces challenges related to data handling and complexity.

Hyperspectral imaging [69] offers the advantage of capturing detailed spectral information across hundreds of wavelengths, making it highly effective for detecting early signs of plant stress and chemical changes. This capability allows its application in a wide range of research fields. However, the high level of detail comes with significant challenges, including the generation of large volumes of complex data that require substantial computational resources for processing. Additionally, the equipment is expensive, and hyperspectral imaging often needs a controlled environment for accurate data collection, making it less practical for commercial use.

In conclusion, each data format technique has its unique strengths and challenges, and the choice of method should be tailored to the specific research objectives, resource availability, and the scale of the grapevine study to ensure the most effective outcomes.

### 6.2. Machine Learning Techniques

In the following paragraphs, we give some brief information about the ML techniques used in the studied papers from an agricultural point of view.

Table 4 illustrates the variety of Machine Learning approaches employed in grapevine research. More specifically, we summarize the Machine Learning techniques used in each disease separately, along with water management, plant deficiencies, and classification. In each of these parts, we reviewed the effective techniques and ranked them as superior (✓) or inferior (✗). In the field “Best ACC”, we retained the highest score of the superior method of each paper by providing some useful information about each study in the “Notes” field. As can be observed, Convolutional Neural Networks (CNNs) and Support Vector Machines (SVMs) are frequently used due to their ability to handle complex data patterns. The accuracy achieved in these studies is generally high, with many approaches exceeding 90%, particularly in disease detection and classification tasks. The notes section suggests that preprocessing steps, data variation, and specific ML models significantly influence the outcomes, highlighting the importance of choosing the right approach for each application.

**Figure 14:**
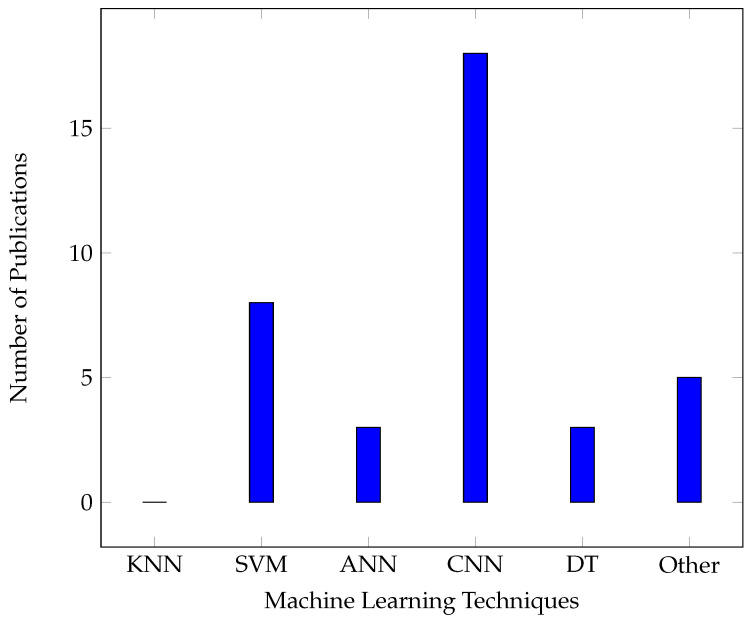
Number of publications identifying each Machine Learning technique as superior.

Additionally, we constructed a bar chart, as shown in Figure 14. This chart illustrates the number of publications where each Machine Learning technique was identified as the most effective or superior method within the studies reviewed. It highlights that Convolutional Neural Networks (CNNs) were the dominant choice, being recognized as the superior technique in approximately 18 publications. Support Vector Machines (SVMs) followed, with around eight publications considering them the most effective. Techniques such as Artificial Neural Networks (ANNs) and decision trees (DTs) were also recognized but to a lesser extent, with about three publications each. The “Other” category includes techniques such as Naive Bayes, Multi-Layer Perceptron (MLP), Radial Basis Function (RBF), etc., which were not individually examined in detail, but overall were considered superior at about five publications. It is worth noting that the K-Nearest Neighbor (KNN) method was included in the analysis, but no publication considered it a superior method; hence, the corresponding bar is practically absent. This chart is intended to provide insight into the relative effectiveness of various Machine Learning techniques as reported in the reviewed literature.

#### 6.2.1. Statistical Measures

Statistical approaches that take into consideration features such as grapevine location, proximity to the ocean, weather, and certain dates are employed in many aspects of grapevine cultivation and wine production. The statistical analysis aids in the early detection and treatment of diseases by detecting disease outbreaks, calculating incidence rates, and studying geographical patterns [79]. It provides knowledge about grapevine growth and the maturity period through phenological investigation. Statistical measures can help determine the impact of climatic and environmental conditions on grapevine health and fruit growth [80].

#### 6.2.2. K-Nearest Neighbor (KNN)

KNN is a Machine Learning algorithm that is used to classify samples based on how near they are to nearby data points. KNN may be used to recognize and categorize sick grapes in a setting of grapevines by comparing their traits to those of surrounding healthy and diseased plants, while closeness can be counted both as a proximity metric or as a comparison. By considering factors such as leaf symptoms, the severity of the disease, and environmental circumstances, KNN can rapidly evaluate the health of grapevines. This approach enables vineyard managers to implement prompt interventions to cut crop losses and implement specialized disease management techniques. The KNN algorithm is a useful tool for improving disease detection in grapevines and enabling proactive management techniques [48,81].

#### 6.2.3. Naive Bayes (NB)

Bayesian methods are well known as a straightforward and efficient supervised learning strategy for accurate and speedy classification tasks. The Naive Bayes classifier is based on the idea that the impacts of other features do not influence the impact of a single feature on a class. This assumption simplifies the algorithm and enables more efficient categorization. Although the independence assumption is not always accurate, Naive Bayes has demonstrated continuous performance in a range of grapevine research applications, being also a popular choice in the field due to its ability to manage large datasets and provide quick forecasts [49].

#### 6.2.4. Support Vector Machine (SVM)

SVMs are well-known supervised Machine Learning algorithms that excel in data classification [49]. The main objective of SVM is to draw a boundary in the data, such as a line or a hyperplane, that effectively divides two groups, thus creating the support vectors. To find the optimum hyperplane, a maximal-margin classifier maximizes the margin, which is given as the perpendicular distance from the border to the support vectors. When total separation is not possible, a soft-margin classifier, which allows some data points to fall between the margins or on the incorrect side of the decision boundary, can be employed [82]. To handle data that are not linearly separable, SVM adds non-linear kernels such as polynomial or radial kernels. The Radial Basis Function (RBF) kernel is widely utilized among the various kernel options due to its adaptability and we have also seen it in agriculture applications. SVM model training, particularly when utilizing the Sequential Minimal Optimization (SMO) technique, has been said to result in exceptionally accurate disease detection models, transforming SVM into a robust non-probabilistic binary linear classifier [51,55,81].

#### 6.2.5. Decision Trees

Decision trees are widely used and helpful in inference and classification procedures due to their simplicity and ease of understanding. They are useful for making predictions since they do not rely on the statistical features of the data [61]. A major problem with Decision trees is overfitting, which results in significantly complicated trees with many layers; to address this, the maximum number of splits is typically limited to decrease the risk of overfitting and maximize the generalizability of the decision tree model [49].

#### 6.2.6. Random Forest

In remote sensing, and especially in viticulture, Random Forest has been employed for various purposes ranging from monitoring water levels in the soil to depicting grapevine water consumption. A Random Forest is an ensemble model of several decision trees constructed using a modified bagging technique. Decision trees are rule-based models that work by continuing to split a dataset into homogeneous groups based on a response variable. Their structure is susceptible to small changes in the supplied data, and ensemble approaches like bagging exploit this trait. Bagging refers to creating several trees from different parts of the original dataset, obtained through resampling approaches. The Random Forest model has been said to perform better than individual learners, through the ensemble, since each tree has a different structure and learns different regions of the dataset. Also, the perturbation process is further enhanced by the fact that trees use just a subset of all available predictors for splitting at each node [35].

#### 6.2.7. C5.0

C5.0 is a robust Machine Learning model that uses a single binary decision tree or a set of rules combined with a boosted approach. It is designed specifically to deal with complex nonlinear interactions, making it useful also in disease detection applications. It has been found to beat other Machine Learning algorithms in some grapevine research, indicating its ability to understand complex data relationships and patterns [37].

#### 6.2.8. Artificial Neural Networks (ANNs)

ANNs (Artificial Neural Networks) have been widely used for classification and regression tasks, with applications also in grapevine research [62]. ANNs seek to mimic the behavior of biological Neural Networks by using connected fundamental components known as neurons that are organized into one or more layers. With a backpropagation method, ANNs change the weights of neurons from the final layer to the first, allowing for efficient learning. In grapevine research, ANNs have been employed for a range of applications such as leaf area index computation [81], rootstock genetics, and disease diagnostics, and it has been also used with hyperspectral data [65].

##### Multilayer Perceptron (MLP)

MLP is a feedforward ANN made up of many layers of connected neurons. A non-linear activation function is used at each neuron to take the weighted sum of its inputs, resulting in complex mapping between input and output data. In grapevine research, the MLP approach has been utilized for disease diagnosis, yield computation, and grapevine classification [83]. Based on input inputs such as images, climatic data, soil conditions, asnd statistical measures, MLP models may give accurate predictions and classifications regarding grapevine health, yield, and disease risk. It is also believed that, by training on historical data, MLP models may identify complex correlations and patterns in grapevine-related data, assisting in better decision-making and sustainable grapevine farming approaches [48].

##### Convolutional Neural Network (CNNs)

Convolutional Neural Networks (CNNs) [45,54] are a subset of ANNs that are widely used in Machine Learning tasks that involve image data, and they have been highly successful in pattern recognition and computer vision tasks. Their structure consists of several types of layers, including convolutional, normalizing, nonlinear, and fully connected layers [57]. CNNs have an advantage in image processing as they can isolate smaller parts of an image, reducing the amount of data being processed at once, while the depth of CNN models allows them to handle nonlinear data. A fully connected layer adapts to the nonlinearity and classifies the information, with the activation function (e.g., ReLU) providing the predicted class label as the output [28,35,50,59]. The UnitedModel for grape leaf disease identification combines GoogLeNet and ResNet architectures, which have been designed to reduce computational costs and address the degradation problem of CNNs, respectively [54,63]. In the case of leaf viral status prediction, a CNN can take an image or a hyperspectral image as input, where each matrix represents the image at different wavelengths, as performed in [46].

##### Single Shot Multibox Detector

SSD (Single Shot MultiBox Detector) is a feed-forward Convolutional Neural Network (CNN) architecture for object detection. It detects objects by employing a specified number of bounding boxes and scores, which are based on a typical Neural Network (NN). The basic modules of SSD are made up of convolutional feature layers that continually diminish in size, allowing the detection of objects of varying sizes. Convolutional filters generate a fixed number of detection predictions per feature map cell, and each feature map cell contains a set of bounding boxes. This design allows for the recognition of objects of varying sizes in images of varying resolutions [53]. Based on the SSD architecture, MobileNets and Inception-V2 are two approaches that have also been used in grapevine research.

- MobileNets are lightweight convolutional neural network models designed to perform Deep Learning tasks on resource-constrained devices. They are attempting to strike a balance between model size and processing efficiency while maintaining acceptable levels of accuracy. MobileNets do this by the use of depthwise separable convolutions, which divide regular convolutions into two different layers: depthwise and pointwise. Depthwise apply a single filter to each input channel and thus minimize computational complexity. Pointwise convolutions employ 1 × 1 convolutions to combine the outputs of depthwise convolutions to capture channel-wise interactions, which results in a reduced number of parameters and calculations required, making it suitable for resource-constrained devices. MobileNets offer flexibility through the use of hyperparameters like width multiplier (α) or resolution multiplier (ρ), which allows for extra trade-offs between model size, processing costs, and accuracy [84].
- Inception-V2 is a deep neural network model designed to deal better with objects that change size across images instead of having to find the optimal kernel. Inception-V2 improves on the original Inception model by reducing computational complexity through the use of factorized convolution techniques. By reducing the number of individual convolutions, Inception-V2 improves runtime performance while maintaining a comparable level of accuracy [85].

#### 6.2.9. The Genetic Algorithm (GA) in Feature Selection

Feature selection aims to identify the most relevant and informative characteristics required for categorization jobs, which is a key stage, particularly in grapevine analysis. The genetic algorithm (GA) is one of the most complex feature selection algorithms [86]. Although GA is a computationally expensive technology, it outperforms traditional selection methods. What separates GA is its ability to handle massive datasets without a prior understanding of the subject under examination. GA explores and manipulates the feature space using natural selection and evolution concepts to find the best subsets of features to contribute to the classification process [42].

#### 6.2.10. Advantages and Limitations of Machine Learning Techniques

This subsection provides a general overview of the advantages and disadvantages of all Machine Learning techniques utilized in the reviewed papers.

K-Nearest Neighbor (KNN) [48,81], while simple and effective for small datasets, becomes computationally intensive during the prediction phase, as it compares new input data to all existing data points. This results in high computational overhead for large datasets, reducing its scalability. Support Vector Machines (SVMs) [51,82] are known for their accuracy and capability to handle high-dimensional data, but they also incur significant computational costs, particularly with large datasets, which can lead to prolonged training times and inefficient processing.

Artificial Neural Networks (ANNs) [62,65] offer flexibility in modeling complex, non-linear relationships within data, making them highly adaptable to various grapevine-related tasks. Similarly, Convolutional Neural Networks (CNNs) [46,50,54], which excel in image-based tasks like disease detection, benefit from their ability to capture intricate patterns in images. However, both ANNs’ and CNNs’ large computational requirements, especially for deeper networks, pose a challenge for real-time applications without adequate computational infrastructure, such as GPUs or cloud computing resources to manage both training and inference efficiently.

Decision trees [49,61] are computationally lightweight and easy to interpret, making them suitable for straightforward applications. Yet, as the model’s complexity increases to handle more intricate datasets, the computational cost also rises. Random Forests [35,52], which are an ensemble of decision trees, offer robust performance and are resistant to overfitting, making them ideal for managing large datasets. However, this ensemble approach amplifies computational demands, particularly during the training process, where multiple trees must be built and optimized.

Naive Bayes [31,49] stands out for its computational efficiency, even when handling large datasets. Its simplicity and ease of implementation make it appealing for certain applications. However, its reliance on the assumption of feature independence limits its effectiveness in scenarios where the features are interdependent, potentially compromising accuracy in more complex datasets.

In summary, each Machine Learning technique presents a trade-off between accuracy, computational cost, and suitability for specific dataset characteristics. Therefore, selecting the most appropriate method for grapevine research must consider these factors to achieve optimal performance in applications such as disease detection and other relevant fields.

## 7. Machine Learning Applications to Grapevine

This section discusses the Machine Learning applications in grapevines by briefly summarizing the 44 papers reviewed in total and separating them according to their field of application in grapevines (diseases, water status assessment, plant deficiencies, and classification) including the geographical location considered in each paper.

**Figure 15:**
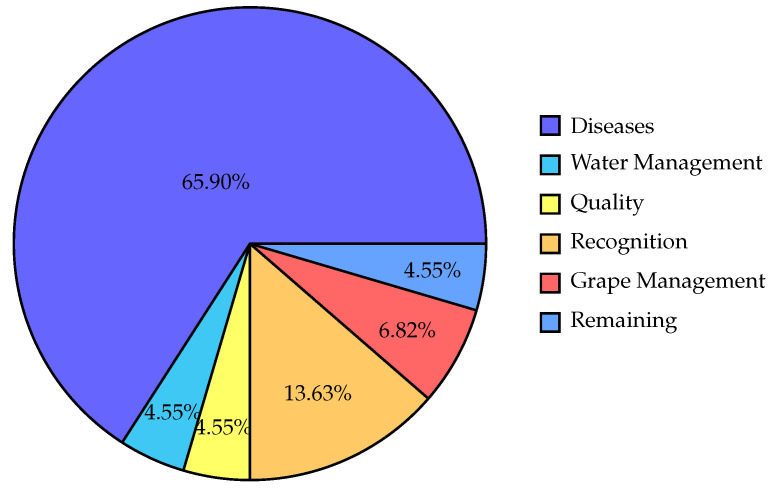
An overview of the reviewed papers according to the field of application.

In Figure 15, we present an overview of the distribution of reviewed papers according to their field of application. In particular, the pie chart reveals a strong emphasis on disease studies, which account for 65.90% of the total. Recognition studies, such as identifying different grapevine varieties or detecting specific characteristics, represent the second-largest category at 13.63%, followed by grape management studies at 6.82%. Quality-related studies, water management studies, and studies classified as “Remaining” each accounted for 4.55% of the total, indicating that these areas were less frequently addressed, so they had smaller contributions in the present bibliographic survey. The “Remaining” studies represent the reviewed papers that do not correspond to any of the other categories.

**Figure 16:**
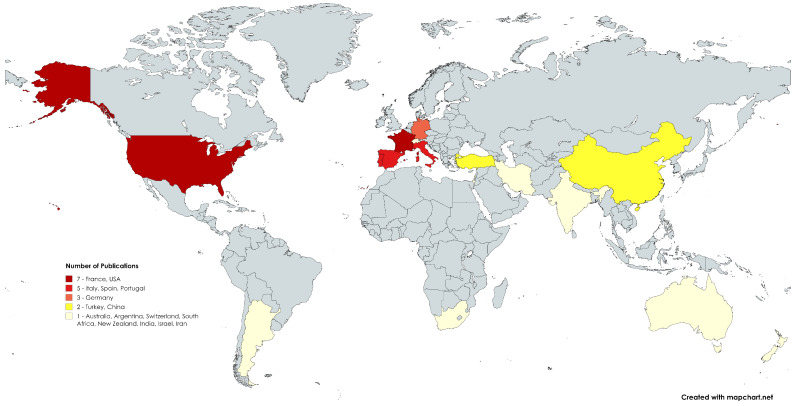
Map of research conducted on vineyards per country.

Moreover, in Figure 16, we overview the geographical locations of each study. In particular, the Figure depicts the geographical distribution of research publications focused on vineyards, revealing that the majority of contributions come from countries, primarily in Europe and the USA, which are ranked as the highest vine producers. The countries are color-coded based on the volume of publications. The darkest red represents countries with the highest number of publications, which are France and the USA with seven publications each. Countries with a slightly lower number of publications (five publications each), such as Italy, Spain, and Portugal, are shown in a lighter shade of red. Germany is represented in orange, indicating a moderate number of publications (three publications). China and Türkiye are shaded in yellow, showing fewer publications (two publications each), while other countries, including Australia, Argentina, Switzerland, South Africa, New Zealand, India, Israel, and Iran, have the least publications one publication each) and are marked in pale yellow. This distribution contrasts with the global landscape of Machine Learning (ML) applications in agriculture, where Asian countries, particularly China and India, dominate, such as in study [7]. In vineyard research, European and US countries play a central role, reflecting their historical and economic ties to viticulture. The map underscores a concentration of vineyard research in traditional wine-producing regions, with relatively limited contributions from other parts of the world, including Africa and much of Asia.

### 7.1. Diseases

Diseases affect grape vines in varying seasons, temperatures, and humidity conditions. For example, in extended hot and humid weather, black rot causes major harm to the grape industry, but it seldom occurs in dry summer. Grape leaf blight is most severe in September when the tree is frail, the temperature is low, and the rain is abundant.

#### 7.1.1. Grapevine Yellow Disease

##### Detection of Grapevine Yellows Symptoms in *Vitis vinifera* L. with Artificial Intelligence

Ref. [18] Location of the experiment/dataset: Tuscany (Central Italy).

This research aims to demonstrate that by using Deep Learning, automatic detection is now achievable, and this can be used for other pests and diseases through transfer learning. Herein, the authors focus on the detection of Grapevine Yellow disease (GY) in red grapevine using Convolutional Neural Networks (CNN) and color pictures of leaf cuttings. They compare six neural network architectures with the best overall accuracy (ACC) of 99.33% obtained by using ResNet-101 followed by ResNet-50, Inception v3, AlexNet, GoogLeNet, and SqueezeNet in that order. There was a concern that the ResNet-101 may have a diminishing return due to its increased complexity, so they concluded that the ResNet-50 provides the optimum balance of accuracy and training cost, which has no significant difference from the ResNet-101. Two data sets are combined for this investigation. The first was from field surveys in Tuscany and the second was from the PlantVillage dataset. These datasets were verified using DNA extraction and real-time PCR tests. Finally, they compare their method to a system that does not employ Deep Learning and to a traditional method of human recognition. They conclude that the Machine Learning system is the best option.

##### Using Image Texture and Spectral Reflectance Analysis to Detect Yellowness and Esca in Grapevines at Leaf-Level

Ref. [39]: Location of the experiment/dataset: Aquitaine, Burgundy (France).

The authors in this research have the purpose of detecting Yellowing and Esca diseases of grapevines utilizing spectral and texture data with the use of Neural Networks as a classification tool to predict the health status of the leaf. In Neural Networks, they used the mathematical model Back Propagation Neural Networks (BPNNs). More specifically, to collect data from infected and healthy leaves, they use texture parameters estimated from red–green–blue (RGB) digital images and hyperspectral reflectance data. As has been observed, the symptoms appear in the leaves during all the periods but mostly in the summer, July and August. In their study, on one side, they execute the empirical PROSPECT model inversion using different models and functions to calculate the biophysical parameters, which are the spectral data. On the other side, they compare how efficient the texture and spectral analyses are in classification. After combining these two datasets, they had an accuracy of 99% for both diseases.

##### Detection of Two Different Grapevine Yellows in *Vitis vinifera* Using Hyperspectral Imaging

Ref. [40]: Location of the experiment/dataset: Middle Rhine (Germany).

The study in this paper is about the Grapevine Yellow disease with its major agents: Bois Noir (BN) and Palatinate Grapevine Yellows (PGY). It was made for data on greenhouse plants of two white grapevine varieties and for data on different red and white varieties that were collected in the field. Disease management is difficult as it does not exist in some treatments. So, it is based on prophylactic measures. The authors use hyperspectral images to detect phytoplasma-infected greenhouse plants and shoots collected in the field. For the differentiation of symptomatic and healthy plants, they tried multiple techniques (LDA, PLS, MLP, rRBF) while also testing in the field and in greenhouses. This had as a result highlighted the true positive and false positive accuracy. Their best accuracy for the classification of the greenhouse plants was 96% with the rRBF model, which was applied to the hyperspectral data recorded for PGY, but the results were worse for Boir Noir (BN). From the shots taken in the field, the classification accuracy ranged from 96% to 100% using the MLP model.

#### 7.1.2. Flavescence Dorée Disease

##### Automatic Detection of Flavescense Dorée Grapevine Disease in Hyperspectral Images Using Machine Learning

Ref. [41]: Location of the experiment/dataset: France, Portugal.

The purpose of this work is the detection of Flavescense Dorée (FD) disease in vineyards using hyperspectral images, and it investigates the use of autoencoders (AEs) with the aim of the dimensionality reduction of these images. Also, the researchers assess the use of the gain-trained encoder as a related feature extractor for the detection of vine diseases. Two methodologies are examined: a patched approach and a full image approach. The dataset comprised 35 hyperspectral leaf images of Vinhão vineyard where 10 samples of them identified with FD. The results showed that with the patched approach, using AEs for the feature extraction of hyperspectral images, the problem of high dimensionality can be faced. For instance, in this case, the patched approach had a 0.2 accuracy increase and a 0.34 increase in AUC, along with an important reduction in computational burden, compared to a common CNN model.

##### Development of Spectral Disease Indices for ‘Flavescence Dorée’ Grapevine Disease Identification

Ref. [42]: Location of the experiment/dataset: Provence-Alpes Côte d’Azur French.

The goal of this work was to create spectral disease indicators (SDIs) for detecting Flavescence Dorée (FD) disease in grapevines. This paper, in particular, identifies disease-specific single wavelengths and wavelength differences using the spectral disease indices (SDIs), combines these specific wavelengths with spectral disease indices, and compares the accuracy of the developed indices to common SVIs. Spectral reflectance was measured in the field under production conditions. Furthermore, unlike previous studies that evaluated just one grapevine variety, this study investigated four distinct grapevine types (two red-berried grapevines and two white-berried grapevines). Finally, it was proved that utilizing vegetative indices was better than using whole spectrum data in most circumstances. In particular, SDIs developed for FD using a genetic algorithm (GA) and with the SVM outperformed typical SVIs with a classification accuracy precision of 96%.

##### Assessment of the Optimal Spectral Bands for Designing a Sensor for Vineyard Disease Detection: The Case of “Flavescence Dorée”

Ref. [43] (continuation of previous research): Location of the experiment/dataset: Provence-Alpes Côte d’Azur French.

This study aimed to define a specialized high-resolution multispectral camera through the exploration of the optimal spectral bands. This camera will be incorporated into an Unmanned Aerial Vehicle (UAV) to identify the parts of the field that are infected with Flavescence Dorée (FD) disease. The benefit of this technique is that it may also be used for other diseases even in a different farming yield. For this spectrometry investigation, the authors used four grapevine varieties (two red and two white) with two spectral analysis strategies to distinguish the healthy leaves from the sick ones. The first method was a feature selection strategy based on the Successive Projection Algorithm (SPA), where some pre-processing techniques were tested and combined with it. The SG1, SG2, and MSC were some of them. The second method focused on Vegetation Indices (VIs) to highlight a specific property of the vegetation. Consequently, two classifiers were employed in this paper: Support Vector Machine (SVM) and discriminant analysis (DA). In general, the SPA approach with the preprocessing technique was better and outperformed common VIs, achieving an overall classification accuracy higher than 96%. The authors conclude that this methodology can be generalized for detecting other plant diseases.

#### 7.1.3. Esca Disease

##### Evaluating the Suitability of Hyper- and Multispectral Imaging to Detect Foliar Symptoms of the Grapevine Trunk Disease Esca in Vineyards

Ref. [44]: Location of the experiment/dataset: JKI Geilweilerhof located in Siebeldingen, Germany.

This research is connected with the previous paper for GY [40]. This study investigates the detection of Esca disease using hyperspectral and multispectral imaging techniques, with field datasets consisting of symptomatic and asymptomatic leaves, over three continuous years from 2016 to 2018. Hyperspectral imaging utilizes a new phenotyping platform called Phenoliner, and multispectral imaging uses a UAV platform, capturing VNIR and SWIR data. In most cases, for VNIR data, the best model was the MLP, and for SWIR, it was the rRBF. The authors achieved the best accuracy of 95% with hyperspectral models regarding annotated field data. This dataset contained the original field data considering only symptomatic leaves. Finally, the authors mentioned that this technology is also a good approach for other disease detection. (the Creative Commons Public Domain Dedication waiver http://creativecommons.org/publicdomain/zero/1.0/ (accessed on 11 August 2024) applies to the data made available in this article unless otherwise stated in a credit line to the data).

##### A Grapevine Leaves Dataset for Early Detection and Classification of Esca Disease in Vineyards through Machine Learning

Ref. [45]; Location of the experiment/dataset: Osimo, Ancona, Marche, Italy.

This article presents a dataset and methodology that could be valuable for researchers who intend to be occupied with the early detection and classification of Esca disease in grapevines using Machine Learning methods. While these methods ensure the early detection of Esca disease, this is helpful for the prevention of its diffusion and for the minimization of financial loss to wine producers. In this study, the dataset includes 1770 images of healthy and unhealthy leaves that are affected by Esca. The Esca disease appears during the July–September period only, so all the images were taken during this period, with three different devices, two smartphones and a tablet. After that, the researchers accreted the data, because this is a useful training technique to increase the diversity of the training set, using the ImageDataGenerator class. In addition, they used a simple CNN architecture for the classification, with the augmentation data used in CNN training, validation, and testing. The augmented dataset was disaggregated as 60% training, 15% validation, and 25% testing. Finally, they achieved an accuracy of 99.96%, 99.57%, and 99.48% in the respective fields.

##### Deep Learning Approach with Colorimetric Spaces and Vegetation Indices for Vine Diseases Detection in UAV Images

Ref. [46]: Location of the experiment/dataset: Region Centre-Val de Loire (France).

In this study, the authors explore a method for diagnosing grapevine diseases based on UAV pictures captured by an RGB sensor combined with Convolutional Neural Networks (CNNs). They used a combination of different patch sizes, color spaces (RGB, HSV, LAB, YUV), and vegetation indices (ExG, ExR, ExGR, GRVI, NDI, RGI) to compare them to CNN performance. The dataset consisted partially of Esca disease. The results demonstrate that CNNs with a mix of ExG, ExR, and ExGR vegetation indices produce the best results, with an accuracy of 95.8%. Also, similar results had the combination of YUV color space with ExGR vegetation indices.

#### 7.1.4. Mildew Disease

##### Predicting Symptoms of Downy Mildew, Powdery Mildew, and Gray Mold Diseases of Grapevine through Machine Learning

Ref. [37]: Location of the experiment/dataset: Tuscany (Italy).

In this paper, the authors conducted research to predict three grapevine diseases using Machine Learning approaches. The diseases that are studied are downy mildew, powdery mildew, and gray mold. To begin with, they collected data from 2006 to 2019, except from 2011. The data of downy and powdery mildew were divided into 80% training and 20% testing (“test1”) from 2006 to 2017, and the data from 2018 and 2019 were tested as separate years (“test2”). For the gray mold disease, only a single test set was used (“test1”) with 80% training and 20% testing within the period from 2006 to 2019. In each dataset, the observations were classified as “inf” for the appearance of disease symptoms or “no” when symptoms were absent. Machine Learning models that were used for comparison were Random Forest (RF) and C5.0. Overall, the C5.0 outperformed RF in sensitivity and balanced accuracy across all the test sets for all three diseases. They achieved in “test 1” a balanced accuracy of 80% for downy mildew, 70% for powdery mildew, and 90% for gray mold. In “test 2”, the prediction accuracy was around 70%. Data on disease symptoms were obtained from Agroambiente.info (http://www.agroambiente.info/, accessed on 13 June 2023).

##### Near Real-Time Vineyard Downy Mildew Detection and Severity Estimation

Ref. [34]: Location of the experiment/dataset: Geneva, NY, USA.

The present study focused on symptomatic downy mildew (DM) infection, which appears as discoloration on the axillary surface of leaves and can be detected without physical contact. Thus, in this research, the authors developed a modified DeepLabv3 network for the near real-time segmentation of DM in high-resolution images, using ResNet18 for efficient multi-scale feature extraction and merging. With the model optimization based on TensorRT developed by NVIDIA to provide high-performance model inference, they achieved with the modified ASPP an optimal balance of speed and comparable accuracy in TensorRT implementations. The dataset was high-resolution images collected using a stereoscopic camera-based stroboscopic light imaging system in the vineyard. The accuracy was measured by the percentage of mean Intersection over Union (%mIoU). Finally, the experimental results showed that the developed model achieved the optimal efficiency accuracy balance of about 83% in DM, supporting effective treatment and precision disease management in the field.

All of the training and testing codes are open-source and available at https://github.com/cu-cairlab/iros2022-OnlineDMSeg.git, accessed on 18 May 2023.

##### Deep Learning for the Differentiation of Downy Mildew and Spider Mite in Grapevine under Field Conditions

Ref. [47]: Location of the experiment/dataset: Spain.

The current study provided a methodology for detecting spider mites and downy mildew on the grapevine and differentiating their symptoms. RGB images of three classes of grapevine leaves were captured with a handheld camera in a commercial vineyard under field and natural light conditions: leaves with downy mildew, spider mite symptoms, and leaves without symptoms. Computer vision techniques (image cropping and GrabCut segmentation in OpenCV) were used to prepare these images for classification. Deep Learning techniques such as data augmentation and CNNs were demonstrated to differentiate the leaf images of the three classes (multiclass classification), achieving accuracy and an F1 score of up to 94%. The researchers additionally used binary classification, which provides the best results in differentiating downy mildew symptoms from spider mite symptoms with a high accuracy and F1 score (89–91%). Even though most studies have focused on a single symptom or different diseases with significantly different visual symptoms, this study tried to make progress by discriminating two important diseases, overcoming the challenge of dealing with symptoms with very similar visual features.

##### Artificial Intelligence and Novel Sensing Technologies for Assessing Downy Mildew in Grapevine

Ref. [48]: Location of the experiment/dataset: Spain.

This research investigates the use of artificial intelligence and non-invasive imaging technologies to assess Downy Mildew (DM) in grapevines, a significant disease that is evaluated under laboratory conditions. The researchers specifically used RGB and Hyperspectral images and applied computer vision and Machine Learning techniques to evaluate the severity of the disease and accomplish early detection. The Machine Learning models used in this research were Convolutional Neural Network (CNN), K-Nearest Neighbor (KNN), MultiLayer Perception (MLP), and Partial Least Square-Discriminant Analysis (PLS). They achieved an accuracy of 82%, using the CNN model with hyperspectral imaging.

##### Early Detection of Grapevine (*Vitis vinifera*) Downy Mildew (Peronospora) and Diurnal Variations Using Thermal Imaging

Ref. [49]: Location of the experiment/dataset: Naan, Road, Israel.

The aim of this study is the early detection of downy mildew disease in grapevines using thermal imaging technology. It is assumed that plant disease generates major changes in leaf temperature. The researchers used a dataset that contained thermographic measurements of the leaves, meteorological measurements gathered concurrently, calculated features from raw data, and manual disease severity evaluation. In addition, five classification models were trained to classify infected and healthy leaves: decision tree, logistic regression, Naive Bayes (NB), Support Vector Machine (SVM), and Ensemble (a combination of Machine Learning techniques), with K-fold cross-validation (K = 5). The best model was an SVM model built on a balanced dataset with cross-validation. The model outperformed all other models evaluated by 10% with 81.6% accuracy, 77.5% F1 score, and 0.874 AUC. They observed that the best time of the day for recording images for downy mildew detection was between 10:40 a.m. and 11:30 a.m., yielding 80.7% accuracy, 80.5% F1 score, and 0.895 AUC.

##### Vine Disease Detection in UAV Multispectral Images Using Optimized Image Registration and Deep Learning Segmentation Approach

Ref. [50]: Location of the experiment/dataset: Centre Val de Loire region in France.

In this work, a novel technique for identifying vine disease utilizing multimodal UAV images (visible and infrared ranges) based on enhanced image registration and a Deep Learning segmentation algorithm was developed. Data were obtained on grapevine plots under actual conditions using the quadcopter UAV drone equipped with two sensors: the visible light sensor (RGB) set and the infrared light sensor (NIR, Red, and NDVI). The method consists of three phases. The first is image alignment, for which an iterative technique based on an interest points detector was created. The second phase separates visible and infrared pictures using a fully convolutional neural network approach (SegNet architecture) to detect four classes: shadow, ground, healthy, and symptomatic vine. Finally, the last phase is to generate a disease map by fusing the segmentations from the visible and infrared images. At the grapevine level, the suggested method obtained more than 92% accuracy of detection at the grapevine level and 87% at the leaf level. One of the disadvantages of this study is the small size of the training sample, which limited Deep Learning segmentation performance.

#### 7.1.5. Leafroll Disease

##### Phenotyping Grapevine Red Blotch Virus and Grapevine Leafroll-Associated Viruses before and after Symptom Expression through Machine Learning Analysis of Hyperspectral Images

Ref. [35]: Location of the experiment/dataset: California, USA.

In this study, the authors tried to use hyperspectral images to classify grapevine leaves as red-blotch- or leafroll-infected or healthy leaves and compared two ML techniques, Random Forest classifier and Convolutional Neural Networks, more specifically 3D-CNN, with RELU and ADAM. Other studies have been conducted using hyperspectral imaging, but their difference relies on the fact that they compare the ease, the techniques used, and the fact that they created a small dataset of 496 images. The dataset created is under laboratory lighting, and one important difference they used in training is that they duplicated images and applied various transformations (rotations, scaling shifts, etc.) to reduce overfitting and amplify the performance of their models. At first, they tried binary classification, but their best results were from a “two-leaf adjustment” method such that if one leaf is classified as infected, both leaves and the plant are classified as infected. Their best accuracy results were 87% and 77.7% for infected vs non-infected leaves and multi-classification, respectively.

##### Early Detection of Grapevine Leafroll Disease in a Red-Berried Wine Grape Cultivar Using Hyperspectral Imaging

Ref. [51]: Location of the experiment/dataset: Prosser, USA.

This research was conducted to examine the potential use of hyperspectral imaging for the non-destructive detection of grapevine leafroll-associated virus 3 (GLRaV-3) during asymptomatic and symptomatic stages of grapevine leafroll disease (GLD). To do so, they used a three-season dataset and pre-processed it to remove outliers as well as extract the feature wavelengths that could be used for classification. For the second part, they used the LASSO technique, while ANOVA was also used to evaluate the sensitivity of feature wavelength; lastly, the LS-SVM classifier was used to evaluate accuracy. After the previous steps, they selected the salient wavelengths (690, 715, 731, 1409, 1425, 1582 nm) and used them to identify GLD-infected leaves during asymptomatic stages with accuracy in stage S1 in the range of 66.67% up to 89.93%.

##### Scalable Early Detection of Grapevine Virus Infection with Airborne Imaging Spectroscopy

Ref. [52]: Location of the experiment/dataset: California, USA.

In this research, they had the idea to use airborne imaging spectroscopy for early detection models of grapevine leafroll-associated virus complex 3 (GLRaV-3). They tried to carry this out by using the NASA AVIRIS dataset while at the same time scouting the grapevines and testing for leafroll disease. The model they used is Random Forest combined with SMOTE. In their research, they used a combination of techniques, including SMR, Savitzky–Golay filter (SG), and PCA before Random Forest with up to 87% accuracy. Also, one observation made is that non-infected and asymptomatic vines have different average reflectance from each other across the spectrum.

Their code is open-source: https://github.coecis.cornell.edu/GoldLab-GrapeSPEC, accessed on 19 August 2023.

#### 7.1.6. Pierce’s Disease

##### Vision-Based Grapevine Pierce’s Disease Detection System Using Artificial Intelligence

Ref. [28]: Location of the experiment/dataset: USA augmented with online images.

This work details a system to detect Pierce’s disease (PD) automatically with images; preliminary results with a prototype Deep Learning system (AlexNet) have a sensitivity of 98.99%. As a dataset, they collected their own data with 597 images of leaves and combined them with control images from plantvillage.org, from publicly available healthy data, and preprocessed them with augmentations. Lastly, they used Deep Learning algorithms, and more specifically Convolutional Neural Networks, and achieved accuracy up to 99% for PD while also training it for black rot disease, Esca, and l eaf spot.

#### 7.1.7. Root Rot Disease

##### Early Identification of Root Rot Disease by Using Hyperspectral Reflectance: The Case of Pathosystem Grapevine/Armillaria

Ref. [31]: Location of the experiment/dataset: Piana Rotaliana, Italy.

In this study, they tried to predict healthy, asymptomatic, symptomatic plants with root rot disease with the use of hyperspectral imaging. They tried five Machine Learning models with the best results given from the Naive Bayes algorithm combined with statistical evaluation beforehand to find sufficient parameters; the accuracy achieved was 90% for healthy vs. diseased and 75% when they included also asymptomatic. They created their own dataset and used ENVI software for analyzing the images and also performed statistical analysis using ANOVA to find variables of interest and use them at a later stage at the classifiers.

#### 7.1.8. General Disease Detection Systems

Some detection systems do not focus on a specific disease but either focus on the classification of multiple diseases or on the general classification of healthy vs. unhealthy.

##### Bringing Semantics to the Vineyard: An Approach on Deep Learning-Based Vine Trunk Detection

Ref. [53]: Location of the experiment/dataset: Portugal.

This study suggests using DL algorithms to detect vine trunks in a rapid and precise way. The main purpose is to compute reliable semantic landmarks to use in Simultaneous Localization and Mapping (SLAM) pipelines of agricultural robots. To achieve this, the authors used a vine trunk dataset called VineSet, which contains RGB images of four different vineyards, and thermal images of a single one, with corresponding annotations for each image. The cameras were contained in their robotic platform AgRob V16. After also using augmentation techniques, the dataset was more than 9000 images. They used the MobileNets and Inception-V2, which are models based on the state-of-the-art Single Shot Multibox Detector (SSD). These models were deployed using an Edge-AI approach and they achieved high frame rate execution. They used some object detection metrics, and the results show that their detectors present an Average Precision of up to 84.16% and an F1 score of up to 84.8%.

##### Automatic Grape Leaf Diseases Identification via United Model Based on Multiple Convolutional Neural Networks

Ref. [54]: Dataset: PlantVillage.

This paper presents a Deep Learning approach to automatically identify grape leaf diseases, i.e., black rot, Esca, and fusariosis leaf spot. The proposed approach is called UnitedModel and is a united Convolutional Neural Networks (CNNs) architecture based on InceptionV3 and ResNet50. The dataset for their study comprises 1619 RGB images of arbitrary size and comes from PlantVillage, which focuses on plant health. They assumed several CNN models and concluded that the UnitedModel is the best on various evaluation metrics such as precision, recall, and average F1-score and achieves a validation accuracy of 99.17% and a test accuracy of 98.57%.

##### Early Detection of Plant Viral Disease Using Hyperspectral Imaging and Deep Learning

Ref. [15]: Location of the experiment/dataset: Columbia, USA.

The potential of employing hyperspectral remote sensing imaging as a non-destructive way to identify grapevines injected with grapevine vein-clearing virus (GVCV) in the early asymptomatic stages was studied in this study. A SPECIM IQ 400–1000 nm hyperspectral sensor (Oulu, Finland) was used to acquire images of each vine. The study aimed to utilize statistical tests to differentiate reflectance spectra between healthy and GVCV grapevines at different stages of infection progression, perform an exploratory analysis to determine the importance of disease-centric vegetation indices, and classify healthy and GVCV grapevines using three approaches, namely vegetation-index-based, pixel-based, and image-based approaches, using handcrafted and automated Deep Learning feature extractors and Machine Learning. The researchers found that reflectance spectra were beneficial in identifying ideal wavelengths for distinguishing between healthy and GVCV-affected vines in the asymptomatic stage. The exploratory research revealed the significance of vegetation indices related to pigment, physiological, and canopy water changes, and the classification performance of the VI-based and pixel-based models was comparable across datasets. The SVM classifier, which is used for prepossessing, performed better in VI-wise classification with smaller feature spaces, but the RF classifier performed better in pixel-wise and image-wise classification with bigger feature spaces. In terms of feature learning from hyperspectral data cubes with a limited number of samples, the automated 3D-CNN feature extractor outperformed the 2D-CNN extractor at the image level, combining with RF and achieving an accuracy of 75%.

##### Entropy-Controlled Deep Features Selection Framework for Grape Leaf Diseases Recognition

Ref. [55]: Dataset: PlantVillage.

The focus of this study was on vine diseases, and it provided a new framework for recognizing and classifying selected diseases in their early stages. The proposed framework included several steps, such as feature extraction, after applying transfer learning to pre-trained deep models using two pre-trained CNN architectures (AlexNet and ResNet101), the selection of the best features using the proposed Yager entropy along with the kurtosis technique (YEaK), and the use of a proposed parallel approach to generate robust feature fusion, which is then classified in one step using five different state-of-the-art classifiers (LS-SVM, Linear SVM (LSVM), QSVM, Cubic SVM (CSVM) and cosine KNN (CKNN). On contaminated vine leaves from the plant village dataset, simulations were conducted, and LS-SVM provided the best classification results with 99% accuracy.

##### Grape Leaf Disease Detection and Classification Using Machine Learning

Ref. [56]: Location of the experiment/dataset: USA.

In this study, the authors present an effective Machine Learning-based approach for the detection and classification of grape leaf diseases. They present four Deep Learning models for grape leaf disease detection and classification based on a developed grape leaf dataset, to generate a comparative analysis and evaluation results to assess their accuracy and performance. These models are Vanilla CNN and the VGG16, MobileNet, and AlexNet with transfer learning. Their dataset includes images from four grape diseases, which are black rot, black measles, leaf blight, phylloxera, and healthy grape plants. After augmentation, the images were more than 5000. They had 97% average accuracy for the selected disease samples and the classification accuracy was 90% for all of the diseases except for the leaf-blight and healthy leaves, which was 100%. The best classification results were achieved by Vanilla CNN with an accuracy of 98%.

##### A Smart Agricultural Application: Automated Detection of Diseases in Vine Leaves Using Hybrid Deep Learning

Ref. [57]: Location of the experiment/dataset: Türkiye/PlantVillage.

This paper describes a study that uses Deep Learning to detect symptoms in vine leaves. The purpose of this research is to improve early disease detection accuracy in vine leaves and to give agricultural engineers a strategy to preserve grape production quality. For this experiment, almost 1000 images of vine leaves from vineyards were gathered. These images were processed using MATLAB 2018B, Deep Learning Toolbox, AlexNet, GoogleNet, and ResNet-18 Convolutional Neural Networks (CNNs). Although CNNs use a normal transfer learning (TL) technique, AlexNet employs a multiclass Support Vector Machine (SVM), while GPU and CUDA are used to speed up the disease diagnosis operation for vine leaves. A software system for the automatic and efficient detection of nine distinct types of leaf diseases, as well as the identification of healthy leaves, was developed. When AlexNet+TL, ResNet-18+TL, GoogleNet+TL, and AlexNet+SVM were used, the overall detection accuracy of this system was 92.5%, 87.4%, 85.0%, and 85.1%, respectively.

##### A Deep-Learning-Based Real-Time Detector for Grape Leaf Diseases Using Improved Convolutional Neural Networks

Ref. [58]: Location of the experiment/dataset: China.

This article proposes a deep-learning-based detector based on improved CNNs to monitor four grape leaf diseases in real time (black rot, black measles/ Esca, leaf blight/leaf disease, and mites), achieving up to 81% accuracy and 15 FPS. To achieve this, they introduced the Inception module and Squeeze-and-Excitation Blocks (SE-blocks) and modified ResNet with the resulting model DR-IACNN, which is said to be faster than the SoA. They also created their own grape leaf disease dataset (GLDD) with combined images from the laboratory and from the grape field. The resulting dataset had 4449 images from different seasons and applied image augmentation to reduce overfitting (rotations, symmetry, Gaussian noise, etc.) which expanded the dataset 14 times.

##### VddNet: Vine Disease Detection Network Based on Multispectral Images and Depth Map

Ref. [59]: Location of the experiment/dataset: France.

In this paper, they presented a new deep learning architecture called Vine Disease Detection Network (VddNet). It uses multispectral images captured from a UAV and trained on a Deep Learning architecture for segmentation designed by the authors named VddNet, which is inspired by other segmentation tools and is based on the VGG encoder. They also created their own dataset using the MAPIR survey2 camera, with a focus on correctly capturing and processing the multispectral images using different algorithms and eventually creating a depth map. They had different accuracy results based on each test, but in all cases, VddNet achieved more than 91% accuracy.

##### UAS-Based Hyperspectral Sensing Methodology for Continuous Monitoring and Early Detection of Vineyard Anomalies

Ref. [60] Location of the experiment/dataset: Douro, Portugal.

This study tries to find diseases, plagues, and other threats capable of putting vines at risk, with the use of a general model trying to perform anomaly detection and lay out a general methodology for early detection of disease using UAS and hyperspectral imaging.

### 7.2. Water Status Assessment

#### 7.2.1. Vineyard Water Status Assessment Using On-the-Go Thermal Imaging and Machine Learning

Ref. [61]: Location of the experiment/dataset: Tudelilla, La Rioja, Spain.

This study presents an on-the-go approach to the estimation of vineyard water status using thermal imaging and Machine Learning algorithms. The dataset they used was created during 7 weeks of summer with three states for each region of interest. A thermal camera was mounted in an all-terrain vehicle, and while there might not be enough data on one hand, on the other hand, there are the times when the vineyards need more water. Two regression models were developed using a combination of rotation forests and decision trees for the development, yielding satisfactory results.

#### 7.2.2. Vineyard Water Status Estimation Using Multispectral Imagery from a UAV Platform and Machine Learning Algorithms for Irrigation Scheduling Management

Ref. [62]: Location of the experiment/dataset: Yunnan, China.

In this research, the authors tried to assess the water status by estimating the midday stem water potential (Psi) of grapevines. The experiment was carried out in a vineyard in the Shangri-La region, located in Yunnan province in China, using multispectral imaging and UAV technology. They created their dataset for testing using a UAV and tried two Machine Learning models to evaluate the correlations between Psi and VIs. One of the models was ANN with 10 layers, while the other was a pattern recognition model ANN. The classification was made between non-stressed vines, moderately stressed, and severely stressed vines, while the second model managed to reach an accuracy of up to 83%.

### 7.3. Plant Deficiencies

Unfortunately, only two studies were found relative to plant deficiencies. A study of the plant deficiencies in various agricultural contexts in [87], and a previous work, especially about grapevine deficiencies, was published in [88]. However, we did not include the latter in our evaluation because our scope was research that was carried out after 2017.

#### A Deep Learning Algorithm for Detection of Potassium Deficiency in a Red Grapevine and Spraying Actuation Using a Raspberry pi3

Ref. [63]: Location of the experiment/dataset: South Africa.

This study used Machine Learning to determine if red grapes contained specific nutrients. Firstly, a different number of iterations of the CNN model were trained, and their accuracies were compared with the SVM model. It was observed that the CNN model outperformed the SVM model, which was proven to be a better choice as a tool to detect the impairments. They tested their particular CNN model with Raspberry Pi 3 and a camera. They processed the images and also evaluated the model using Python’s OpenCV library. The SVM model was trained on just a few images and made use of the HOG scalar descriptor, the radial RBF function, and principal component analysis.

### 7.4. Classification

The literature provides so many classifiers that it is very difficult for researchers to find the perfect model for certain tasks, such as classifying agricultural-related objects, or grapevine varieties. An intelligent solution is to try out many classifiers and finally choose one with the highest prediction accuracy, as mentioned in [42].

#### 7.4.1. In-Field High Throughput Grapevine Phenotyping with a Consumer-Grade Depth Camera

Ref. [64] Location of the experiment/dataset: Switzerland.

This research presents an in-field high throughput grapevine phenotyping platform based on an Intel RealSense R200 depth camera placed on the back of an agricultural vehicle. There were two issues addressed: canopy volume estimate and grape bunch detection. As an interesting idea, they used a 3D model to represent the environment before starting the classification. They made classifications for different parts of the grapevine and external factors, specifically the background, leaves, wood, pole, and bunch. In addition, four Deep Learning frameworks (AlexNet, VGG16, VGG19, and GoogLeNet) were used to classify visual pictures obtained by the RGB-D sensor and distinguish grape bunches. Despite the poor quality of the input images, all techniques properly identified fruits, with a maximum accuracy of 91.52% attained by the VGG19.

#### 7.4.2. A CNN-SVM Study Based on Selected Deep Features for Grapevine Leaves Classification

Ref. [36]: Location of the experiment/dataset: Türkiye.

Deep-Learning-based classification is used in this study on images of grapevine leaves. Images of 500 vine leaves from five different species were obtained for this purpose using specific self-illuminating equipment. The classification was carried out using a cutting-edge CNN model fine-tuned MobileNetv2. The second strategy retrieved features from the pre-trained Logits layer of MobileNetv2 and classified them using different SVM kernel functions, i.e., CNN-SVM structure. The third technique selected and classified features collected from the Logits layer of the MobileNetv2 model using the Chi-Square method. Cubic was the most successful SVM kernel. The system’s classification success rate has been determined to be 97.60%. Even though the number of features used in classification decreased, feature selection improved classification success.

#### 7.4.3. On-the-Go Hyperspectral Imaging under Field Conditions and Machine Learning for the Classification of Grapevine Varieties

Ref. [65]: Location of the experiment/dataset: Logroño, La Rioja, Spain.

This research presents a unique method for classifying a large number of grapevine (*Vitis vinifera* L.) cultivars in the field using hyperspectral in-motion images and a range of Machine Learning algorithms. The dataset was created by mounting a hyperspectral camera to an all-terrain vehicle and imaging in natural light while on the go. Spectra were collected on the canopy of 30 different cultivars in a commercial vineyard during two distinct leaf phenological phases. With the purpose of testing with alternative algorithm settings and spectral preparation processes, a large number of models were generated using Support Vector Machines (SVMs) and artificial Neural Networks (multilayer perceptrons, MLPs). Both classifiers worked magnificently, allowing them to train models with F1 recall scores and area values under receiver operating curves at an order of up to 0.99. The most significant addition of this work was that no previous research had been carried out on the classification of plant varieties in the field, either on-the-fly or by ground-based hyperspectral imaging.

#### 7.4.4. Image Classification for Detection of Winter Grapevine Buds in Natural Conditions Using Scale-Invariant Features Transform, a Bag of Features, and Support Vector Machines

Ref. [66]: Location of the experiment/dataset: Lujan de Cuyo branch, Mendoza, Argentina.

This study describes a method for classifying images of grapevine buds with a diameter of 100 to 1600 pixels taken outdoors, in a natural environment, in winter (i.e., without grapes, with few leaves and dormant buds), with no artificial background and with the minimum number of tools. The recommended method uses well-known computer vision technologies such as scale-invariant feature transformation to generate low-level features and Support Vector Machines to train the classifier. When testing images with buds of at least 100 pixels in diameter, the method achieves a recognition rate of more than 0.9 and an accuracy of 0.86 for windowed regions covering the entire bud and up to 60% of it, scaled to the windowed areas containing a proportion of 20–80% bud versus background pixels.

#### 7.4.5. Automated Grapevine Cultivar Identification via Leaf Imaging and Deep Convolutional Neural Networks: A Proof-of-Concept Study Employing Primary *Iranian varieties*

Ref. [67]: Location of the experiment/dataset: Malayer, Iran.

This paper presents a convolutional neural network (CNN) framework for identifying grapevine cultivars by using leaf images in the visible spectrum (400–700 nm) and achieving 99% accuracy. Their difference is that instead of focusing on classification between different species, they focus on classifying different variants of the same species. To achieve this, they created their small dataset to classify six grapevine cultivars in Iran and also used the imageNet dataset for fine-tuning. As key points in their model, they used RELU, SoftMax, and Adam optimizer, while for their architecture, they modified VGG16 by replacing the last three dense layers with a classifier block.

### 7.5. Others

#### 7.5.1. Geographical and Cultivar Features Differentiate Grape Microbiota in Northern Italy and Spain Vineyards

Ref. [89]: Location of the experiment/dataset: Northern Italy, Italian Alps, Northern Spain.

The work is mainly from the biological perspective and explores the grapevine microbiome and its relationship with geographical origin and cultivar. The study used high-throughput sequencing to analyze the microbiomes of three grape cultivars from different regions in Northern Italy and Spain. It found that although specific bacterial taxa are shared in all the samples, specific microbial signatures may point to geographic origin and cultivar. The study further supported the claim that the main source of grape-associated bacteria is vineyard soil and showed the possibility of using Machine Learning to predict grape origin and cultivars with a high degree of accuracy from microbiome composition at a smaller scale.

#### 7.5.2. Detection of Single Grapevine Berries in Images Using Fully Convolutional Neural Networks

Ref. [90]: Location of the experiment/dataset: Siebeldingen, Germany.

This study employs fully Convolutional Neural Networks to examine images captured from the field phenotyping system, Phenoliner. More specifically, they counted grapevine berries in an attempt to improve the identification of clusters. By reframing instance segmentation to semantic segmentation through the differentiation of classes berry and edge, they could accurately count and locate berries in the cluster for phenotypic analysis, berry size, for instance. The research on the analysis of 60 images, limited to Riesling plants on several trellis constructions, exhibited an encouraging accuracy of 94.0% for vertical shoot positioned trellis and 85.6% for semi-minimum pruned hedges.

#### 7.5.3. Insect Classification and Detection in Field Crops Using Modern Machine Learning Techniques

Ref. [91]: Location of the experiment/dataset: Tiruchirappalli, Tamil Nadu, India/Deng, IP102 datasets.

This study was a case study on classifying and identifying various insect datasets using insect detection methodology and Machine Learning, as well as analyzing the results achieved. However, it is impossible to classify insects accurately in real-time agricultural conditions because of factors such as shadows and foliage. The study considers a method of using several Machine Learning algorithms, where the model CNN recorded the highest classification accuracy of 91.5% and 90% for 9 and 24 classifications of the insects in the Wang and Xie datasets, respectively. As such, the increased accuracy helped to reduce the time taken during computation for insect classification. The insect pest detection system also classified insects well across several datasets with less processing effort.

#### 7.5.4. Path Planning Algorithms Benchmarking for Grapevines Pruning and Monitoring

Ref. [92]: Location of the experiment/dataset: Douro, Portugal.

This work benchmarks six algorithms from the open manipulation planning library when using a cost-effective six-degree freedom manipulator operating in the simulated vineyard, selecting the most difficult path planning case, the pruning task, to evaluate the performance of all the path planning algorithms. In general, the OMPL planners showed low performance under taxing pruning. The most favorable and future-looking data were collected and acquired by the BiTRRT algorithm. This benchmark allows the reader to understand the ability of each of the algorithms and its best operating environment.

#### 7.5.5. A Robot System for Pruning Grape Vines

Ref. [93] Location of the experiment/dataset: New Zealand.

The present paper presents a robot system for the automatic pruning of grapevines. Over the row of vines moves a mobile platform equipped with a trinocular stereo camera to image the vines along its path. A three-dimensional model of the vines was constructed using a computer vision system. An artificial intelligence design is used to determine which canes to prune from the vines. The robot arm with six degrees of freedom finally makes the necessary cut.

#### 7.5.6. Assessment of Smoke Contamination in Grapevine Berries and Taint in Wines Due to Bushfires Using a Low-Cost E-Nose and an Artificial Intelligence Approach

Ref. [94]: Location of the experiment/dataset: Australia.

This paper is about using e-nose sensors on wines to detect bushfires as well as assisting vintners in alleviating smoke taint. E-nose measurements were taken as input in deriving a classification model through Machine Learning algorithms with seven neurons targeting the treatments. The winemakers could utilize these models, which enabled them to know the levels of smoke contamination in near real time and develop measures for the alleviation of smoke taint in wines after bushfires.

#### 7.5.7. VineInspector: The Vineyard Assistant

Ref. [95] Location of the experiment/dataset: Vila Real, Portugal.

In this study, VineInspector is a tool that helps monitor conditions, collect images with cameras, and detect prevalent pests and diseases. This method utilizes modern artificial intelligence and computer vision techniques to recognize, categorize, and discern traits in captured images over time, which are accessible via an Internet of Things (IoT)-enabled cloud platform. The VineInspector is developed, tested, and field-validated for accuracy and functionality, automating tasks previously performed by viticulturists, including the assessment of grapevine shoot size and the counting of pests. This will make the monitoring of health in vineyards more efficient and responsive to pests and diseases. One example is to counter mildew.

## 8. Discussion and Conclusions

The study provided a comprehensive review of artificial intelligence and Machine Learning applications in grapevine research, underscoring the necessity of innovative solutions in contemporary viticulture. Considering the enhanced challenges related to climate change, pests, and diseases and increased demands for better-quality grapes, AI, more precisely ML, plays a very important role in optimizing grapevine production management. The review focused on 44 studies in total conducted between 2017 and 2023, summarizing the research works made in that period by comparing various datasets, diseases, ML techniques, and data formatting techniques and their applications in viticulture. In summary, the following major conclusions can be drawn.

1. A key finding of this review is the predominant use of Neural Networks, especially Convolutional Neural Networks (CNNs), in the majority of reviewed papers, as can be seen in Table 4 and the bar chart drawn in Figure 14, which had the best results in most cases for the image-based tasks like grapevine disease detection and classification, as well as in other applications such as water management and plant nutrition. Besides CNNs, other ML techniques such as Support Vector Machines, decision trees, and Random Forests, among others, were considered, which contribute to the enhancement of accuracy and efficiency especially in applications such as the prediction for grapevine diseases and grapevine classification.
2. Given the scarcity of open, standardized datasets, we have acknowledged the significance of datasets in Machine Learning (ML) applications, particularly in grapevine research, which often necessitates researchers to develop their own. Progress has been achieved in this area, with increased open data related to diseases and classification purposes. Section 5 provided an exhaustive list of available datasets, outlining the types of data they encompass, including RGB images, hyperspectral and multispectral imaging, and thermal data. These datasets play a pivotal role in training ML models for detecting and predicting diseases such as Grapevine Yellow, Esca, downy mildew, and leafroll, among others. In addition, by reviewing existing datasets related to grapevine research, the paper identifies data gaps, which could help researchers draw important conclusions about trends for future data collection and how these datasets could be merged or supplemented.
3. The review also explored the utilization of advanced imaging techniques, shown in Table 2, such as Unmanned Aerial Vehicles (UAVs or drones), which exhibit high-resolution data acquisition capabilities. Although minimal research has been conducted on the use of thermal images for grapevine management, UAVs and imaging approaches, such as hyperspectral imaging, have been extensively employed by researchers, yielding promising results.
4. The study compared AI techniques in grapevine research, such as computer vision, Deep Learning, and Machine Learning, assessing their effectiveness in different contexts. As shown in Figure 15, a significant portion of the research (about 65.9%) focuses on disease-related topics, where we contrast methods of identification and prediction of grape infections based on symptoms, environments, or other factors. This helps in the early diagnosis of different diseases, enabling early pest management, and facilitates researchers and grape growers in choosing the best techniques for their particular needs, hence improving crop health and productivity. Conversely, areas like water management and grape quality receive less attention (about 4.55%), indicating theunder-exploration or reliance on more generalized agricultural methods.
5. Comparisons in this area should be made with extreme caution because several factors affect the reported accuracy. The most important are the datasets used, the data format techniques used to capture them, the different geographic locations tested, and how general the algorithms are, e.g., how effective they are at making accurate predictions or decisions when they encounter new information. Furthermore, through the review of the 44 papers, it has been observed that if a dataset contains only one disease, it is easier to obtain models with better performance compared to techniques applied to datasets with multiple diseases.
6. As can be inferred from the geographical distribution, depicted in Figure 16, combined with the wide range of research fields, the applications of ML to facilitate various aspects of management in the wine sector is an important topic on an international scale. In particular, the geographical distribution of research publications focusing on the vineyard reveals that the majority of contributions come from countries in Europe and the USA, while in contrast, fewer studies come from countries such as China and South Africa, among others. This finding contrasts with the global landscape of Machine Learning (ML) applications in agriculture, where Asian countries, particularly China and India, dominate, such as in the study [7], highlighting that in vine research, the European and US countries play a central role, reflecting their historical and economic ties to viticulture.

In conclusion, this study identifies how more powerful algorithms are needed to handle the variability and complexity of agricultural data, and it is also important to have greater availability of integrated datasets. Moreover, it highlights the potential of AI and ML on grapevines and viticulture research, and more research in unexplored areas such as soil management and crop quality is encouraged. In addition, valuable information is offered for researchers, agricultural scientists, and viticulturists. Finally, the methods based on AI can significantly contribute to more successful and economically sustainable grape production by enhancing early disease detection.

## Figures and Tables

**Table 1:** The “Datasets” column references the datasets mentioned below. The “Diseases” columns depict the number of raw images used in each dataset for each disease. The “Class” column contains image data used for classification. The “Info” column includes some comments for each dataset.

Datasets	Diseases						Class	Info
	Healthy	GY	Esca	Mildew	Leafroll	Others		
1							2078	Grape Varieties + Chemical Data).
2	423		1383			1180 + 1076		Black rot + Leaf-blight.
3	220		240			210 + 2010		Leaf-blight + Black rot.
4				282				Mentioned the total number of images regardless of healthy or diseased).
5	84	134	1383			1180 + 1076		Expanded version of 2.
6	881		887					-
7	135				156	108 + 97		Red Blotch images and combined Red Blotch and Leafroll images.
8								Statistical Data.
9			107			228	1483	228 Flavescence Dorée images + 5 Grape varieties.

**Table 2:** Depiction of how data are acquired, and in what format, for each reviewed paper with the corresponding application.

		Cite	Data Format Techniques					Notes
			RGB	Spectral		Thermal	UAV	
				Hyper	Multi			
DISEASE	GY	[18]	✓					
		[39]	✓	✓				PROSPECT model.
		[40]		✓				
	DOREE	[41]		✓				Autoencoders—dimensionality reduction.
		[42]			✓			
		[43]			✓			
	Esca	[44]		✓	✓			VNIR and SWIR cameras (from RGB).
		[45]	✓					ImageDataGenerator.
		[46]	✓				✓	
	MILDEW	[37]						Geographical data analysis (statistics).
		[34]	✓					
		[47]	✓					
		[48]	✓	✓				
		[49]				✓		Meteorological data.
		[50]			✓		✓	
	LEAFROLL	[35]		✓				Laboratory lighting.
		[51]		✓				
		[52]			✓			Airborne imaging (NASA AVIRIS).
	PIERCE’S	[28]	✓					
	Root Rot	[31]		✓				
	GENERAL	[53]	✓			✓		
		[54]	✓					
		[15]		✓				
		[55]	✓					
		[56]	✓					
		[57]	✓					
		[58]	✓					
		[59]			✓		✓	
		[60]		✓			✓	Anomaly detection.
WATER—STATUS		[61]				✓		
		[62]			✓		✓	
PLANT DEFICIENCIES		[63]	✓					Raspberry Pi 3.
CLASSIFICATION		[64]	✓					3D Depth Camera.
		[36]	✓					
		[65]		✓				In motion images.
		[66]	✓					In natural conditions.
		[67]	✓					

**Table 3:** Pros and cons of data formatting techniques in ascending order based on the estimated cost of each technique.

Data Format Technique	Pros	Cons
RGB Images	+ High spatial resolution. + Well-suited for visual analysis. + Simple to train models. + Requires no expert knowledge. + Compatible with existing equipment.	− Limited spectral information. − Can miss non-visible features. − Hard for detection of asymptomatic leaves or early detection applications.
Thermal Images	+ Useful for detecting plant stress and water status. + Low cost. + Can be combined with other techniques. + Adds one more useful parameter.	− Sensitive to environmental conditions. − Limited to surface temperature data. − Difficult to apply independently in other fields.
Unmanned Aerial Vehicle (UAV) Images	+ Allows large area coverage. + Can combine multiple imaging techniques. + Can be automated. + Faster to scan the whole field. + Can be used daily leading to faster detection of issues. + Expensive to purchase, but reduces cost over time.	− Skill requirements. − Requires flight permits. − Regulations vary by country. − Limited flight time depending on the battery. − Weather dependency.
Multispectral Images	+ Balances between spatial and spectral data. + Can be used in multiple applications. + Lower cost than Hyperspectral imaging, having comparable results.	− Less detailed than hyperspectral imaging. − Moderate computational demand. − Complexity. − Data handling.
Hyperspectral Images	+ Provides detailed spectral information. + Can be used in even more applications. + Too much information for the researchers.	− High data complexity. − Expensive equipment required. − Needs specific environment and data handling. − Hard for commercial use.

**Table 4:** Representation of Machine Learning techniques utilized in each reviewed study, with the corresponding best accuracy.

		Cite	Machine Learning Techniques						Best ACC	Notes
			KNN	SVM	ANN	CNN	Decision Trees	Other		
DISEASE	GY	[18]				✓			99.33%	ResNet101.
		[39]			✓				99%	Back Propagation Neural Network.
		[40]						rRBF, MLP	96%	Varies between tests (e.g., field, BN-GY).
	DOREE	[41]				✓			83%	
		[42]		✓					96%	
		[43]		✓					96+%	
	Esca	[44]						rRBF	95%	Varies depending on the type of data and the year.
		[45]				✓			99.48%	
		[46]				✓			95.8%	
	MILDEW	[37]					✗	C5.0	70+%	Better Accuracy when tested diseases separately.
		[34]				✓			83%	Modified ResNet.
		[47]				✓			94%	
		[48]	✗			✓			82%	
		[49]		✓			✗		81.6%	
		[50]				✓			92+%	SegNet Architecture.
	LEAFROLL	[35]				✓	✗		87%	3D-CNN, Binary classification on a symptomatic dataset.
		[51]		✓					89.93%	Least-squares SVM.
		[52]					✓		87%	Random Forest.
	PIERCE’S	[28]				✓			99.2%	AlexNet Architecture.
	Root Rot	[31]						NB	90%	Healthy vs Diseased plants.
	GENERAL	[53]				✓			84.16%	Average precision, MobileNets, Inseption-V2.
		[54]				✗			99.17%	UnitedModels based on 4 models.
		[15]		✓		✓	✓		75%	Preprocessing: SVM, Prediction: 3D-CNN, RF.
		[55]	✗	✓					99%	LS-SVM, cosine KNN, several steps from preprocessing to prediction.
		[56]				✓			98%	Vanilla CNN 100% accuracy with ensemble model.
		[57]				✓			92.5%	AlexNet + Transfer Learning.
		[58]				✓			81.1%	Focused in speed and multiple Disease Detection.
		[59]			✓				93.72%	VddNet.
		[60]							-	
WATER—STATUS		[61]					✓		65%	Regression models, $R^{2}$ for prediction.
		[62]			✓				82.9%	Pattern recognition ANN.
PLANT DEFICIENCIES		[63]		✗		✓			80%	
CLASSIFICATION		[64]				✓			91.52%	VGG19.
		[36]		✓		✗			97.6%	Cubic SVM.
		[65]		✗				MLP	99%	
		[66]		✓					90.8%	
		[67]				✓			99.11%	Modified VGG16.

## Data Availability

Data are contained within the article.
